# Therapeutic effects of herbal-medicine combined therapy for COVID-19: A systematic review and meta-analysis of randomized controlled trials

**DOI:** 10.3389/fphar.2022.950012

**Published:** 2022-09-01

**Authors:** Tsai-Ju Chien, Chia-Yu Liu, Yuan-I Chang, Ching-Ju Fang, Juo-Hsiang Pai, Yu-Xuan Wu, Shuoh-Wen Chen

**Affiliations:** ^1^ Division of Hemato-Oncology, Department of Internal Medicine, Branch of Zhong-Zhou, Taipei City Hospital, Taipei, Taiwan; ^2^ Institute of Traditional Medicine, National Yang Ming Chiao Tung University, Taipei, Taiwan; ^3^ Department and Institute of Physiology, College of Medicine, National Yang Ming Chiao Tung University, Taipei, Taiwan; ^4^ Medical Library, National Cheng Kung University, Tainan, Taiwan; ^5^ Department of Secretariat, National Cheng Kung University Hospital, College of Medicine, National Cheng Kung University, Tainan, Taiwan

**Keywords:** herbal medicine, COVID-19, systematic (Literature) review, meta-analysis, complementary therapy, traditional chinese medicine

## Abstract

**Background/Aim:** Since 2019, the COVID-19 pandemic has been a devastating disease affecting global health to a great extent. Some countries have added on herbal medicines as a complementary treatment for combating COVID-19 due to the urgency of stopping the spread of this viral disease. However, whether these herbal medicines are effective is uncertain. This systematic review and meta-analysis aimed to evaluate the effects of herbal medicine combined therapy in the treatment of COVID-19.

**Methods:** A literature search was performed following the PRISMA Statement and without language restrictions. Seven databases were searched from inception through December 2021. All selected studies were randomized clinical trials (RCTs). Comparing the effects of herbal medicine combined therapy with conventional western medicine, including improvement of clinical symptoms, chest CT images, viral conversion rate, C-reactive protein (CRP) and interleukin 6. Cochrane criteria were applied to examine the methodological quality of the enrolled trials; and meta-analysis software (RevMan 5.4.1) was used for data analysis.

**Results:** In total, the data of 5,417 participants from 40 trials were included in this systematic review; and 28 trials were qualified for meta-analysis. The trials had medium-to-high quality based on GRADE system. Meta-analysis showed that combining herbal medicine vs conventional treatment in 1) coughing (1.43 95% CI:1.21, 1.71, *p =* 0.0001), 2) fever (1.09 95% CI:1.00, 1.19, *p =* 0.06), 3) fatigue (1.21 95% CI:1.10, 1.33, *p =* 0.0001); 4) CT images (1.26 95% CI:1.19, 1.34, *P* ≤ 0.00001), 5) viral conversion rates (1.22 95% CI:1.06, 1.40, *p =* 0.005) and 6) viral conversion times (−3.72 95% CI: −6.05, −1.40, *p =* 0.002), 7) IL6 change (1.97 95% CI: −0.72, 4.66, *p =* 0.15) and 8) CRP change (−7.92 95% CI: −11.30, −4.53, *P* ≤ 0.00001).

**Conclusion:** Herbal medicine combined therapy significantly reduces COVID-19 clinical symptoms, improving CT images and viral conversion rates. Reported adverse events are mild. However, for certain biases in the included studies, and the need for further study on effective components of herbal medicine. Further large trials with better randomized design are warranted to definite a more definite role of herbal medicine.

## 1 Introduction

Since December 2019, the outbreak of the COVID-19 pandemic has had devastating effects on global health systems and economic growth, and has affected the lifestyle of human populations on a large scale (Sreepadmanabh et al., 2020). In March 2020, the World Health Organization (WHO) declared that COVID-19 was a global pandemic because the viral infection had spread rapidly within a growing number of countries.

The causative agent of COVID-19, severe acute respiratory syndrome coronavirus 2 (SARS-CoV-2), distressed not only the respiratory tract system (Zhu et al., 2020), but also presented as a systemic disease associated with vascular inflammation; this affected multiple organs and eventually led to multi-organ failure in severely affected individuals (Ding et al., 2020; Smadja et al., 2021). Therefore, it became urgent to prevent or treat COVID-19 early and effectively.

Currently, treatment of COVID-19 includes administering antiviral and symptomatic support in mild cases; whereas no definitively effective drugs are available to treat this viral infection even though some non-specific therapeutic options exist and vaccination is ongoing (Mirtaleb et al., 2021). Since August 2021, the delta variant has exhibited high capability of invading the host’s immune system and appears to be more transmissible than other variants, making people more anxious (Harder et al., 2021; Shiehzadegan et al., 2021). In November 2021, the appearance of the Omicron (B.1.1.529) variant expressed high enhanced transmissibility and immune evasion and was shown to re-infect individuals previously infected with other SARS-CoV-2 variants (Kannan et al., 2021). In this scenario, beyond the conventional western medicine (CWM) approach, which includes antiviral drugs such as interferon, ribavirin, lopinavir-ritonavir, and chloroquine phosphate (Negrut et al., 2021; Wong et al., 2021), there is widespread use of antibacterial drugs, antitussives, expectorants and supportive therapy, while Herbal -medicine combined therapy is also flourishing in many countries. Since the origin of the COVID-19 outbreak in China in 2019, herbal medicine (HM) has been used to help combat COVID infection along with modern anti-infective agents because no vaccine or definitive antiviral treatment was available for COVID-19 at that time. In Asian countries such as China, Taiwan and Hong-Kong, HM has played a critical role in both treating and preventing COVID-19; the inclusion of traditional Chinese medicine (TCM) in the Chinese protocol is based on its long, successful historic experience in fighting against pestilence (Ni et al., 2020). To have a successful experience combating COVID-19, the TCM therapeutic schedule was included in the guidelines for diagnosis and treatment of COVID-19 (Jin Y. H. et al., 2020). Broadly speaking, many other countries such as India and Japan have also applied HM to alleviate the effects of infectious diseases in the context of SARS-CoV-2 (Ang et al., 2020a; Ang et al., 2020b). The volume of existing reports and systematic reviews provide irrefutable evidence that HM combined therapy possesses a potential antiviral capability against SARS-CoV-2 (Fan et al., 2020; Du et al., 2021), yet the lack of solid evidence-based trials makes the value of herbs vague (Panyod et al., 2020). Furthermore, no grading was performed on the certainty of evidence for their results also easily skew the result directions if not handled properly.

In addition to herbal medicine, some natural products were also under investigation, more and more studies applied network pharmacology in exploring the related pathways and mechanisms of these herbs and natural compounds (Fan et al., 2020; Ang et al., 2021). The more research focus on this field, the more appealing and potential of these herbs were found.

To date, many HM-related trials (not only TCM) have been published on aspects of COVID-19; however, the high heterogeneity and loose trial design made it hard for previous studies to draw definitive conclusions. To further understand the efficacy of HM combined therapy in treating the COVID-19, we conducted a systematic review and meta-analysis to evaluate the efficacy of HM in the treatment of COVID-19 objectively.

## 2 Methods

The protocol for this review and meta-analysis has been registered on the International Prospective Register of Systematic Reviews (PROSPERO) with the registration number CRD42021287021. This review was reported according to the updated Preferred Reporting Items for Systematic Reviews and Meta-Analyses (PRISMA) (Page et al., 2021).

### 2.1 Data sources and search strategy

This systematic review was conducted in compliance with the PRISMA Statement ensure transparent and complete reporting. The following 7 databases were searched for relevant randomized clinical trials, with no language restrictions, from their inception dates to 30 December 2021: Embase (Elsevier), Medline (Ovid, including epub ahead of print, in-process, and other nonindexed citations), Cochrane Library (including clinical registers from WHO ICTRP and US ClinicalTrials.gov), CINAHL Complete (EBSCOhost), Scopus, China National Knowledge Infrastructure (CNKI) and Wanfang Data. The reference lists of eligible articles were also reviewed to identify additional studies for possible inclusion. We also manually retrieved relevant studies and clinical trials to acquire as many studies as possible. E-mail alerts were established to identify newly released studies from the different databases that fell within the scope of our review.

The key concepts – COVID-19 and Traditional Chinese Medicine–used in the search included their 216 synonyms in total and controlled vocabulary (12 Emtree terms, 13 MeSH terms, etc.). Highly sensitive search filters were applied to identify randomized clinical trials. Supplementary Appendix A1 displays the full search strategy for the individual databases.

### 2.2 Eligibility criteria and data extraction

All eligible studies examined studies that fulfilled the inclusion criteria, as follows: 1) Studies designed as randomized clinical trials (RCTs); 2) Adult patients (aged 18 years and older) with an established diagnosis of COVID-19 in evaluable status. The criteria of mild and moderate is according to the Clinical Spectrum of SARS -CoV-2 Infection from National Institutes of Health(Maier et al., 2021), which set mild illness as individuals who have any of the various signs and symptoms of COVID-19 (e.g., fever, cough, sore throat, malaise, headache, muscle pain, nausea, vomiting, diarrhea, loss of taste and smell) but who do not have shortness of breath, dyspnea, or abnormal chest imaging; and moderate illness as individuals who show evidence of lower respiratory disease during clinical assessment or imaging and who have an oxygen saturation (SpO2) ≥94% on room air at sea level. 3) The intervention group was treated with HM combined therapy. Patients in the control group were required to be treated with CWM or a combination of HM placebo and CWM. We excluded studies designed as retrospective studies, observational studies, repeated data studies and cross-sectional studies. Studies which set outcomes only with TCM syndromes evaluation, sample size less than 30; or if the full text cannot be obtained were also excluded. The selection of studies and data extraction were performed independently by two reviewers (Chien and Liu) according to the inclusion and exclusion criteria.

### 2.3 Risk of bias and quality assessment

Two reviewers (Chien and Liu) assessed the methodological quality of studies by using the Cochrane Collaboration’s tool (Higgins et al., 2022)and the new version of this tool, Risk of Bias version 2 (RoB 2). Six items of ROB 2 were evaluated as follows: Randomization, Deviations from intended interventions, Missing outcome data, Measurement of the outcome, Selective Outcome reporting, overall bias (Cochrane Collaboration, 2021). Once the disagreements were noted, discussions were held with the other investigators (Chang; Wu) to make a consensus decision.

### 2.4 Assessment of evidence certainty

We assessed the outcomes by using GRADE methodology (Guyatt et al., 2008). The overall evidence certainty was evaluated by using five downgrading domains which included considerations of study limitation, inconsistency, indirectness, imprecision, and publication bias. The level of evidence was classified as high, moderate, low, or very low. Grading was performed using GRADE pro software (Diekemper et al., 2018) (available from http://www.gradepro.org).

### 2.5 Outcome measures assessment

In the review, the outcome measures in the enrolled studies included chest CT scan; blood tests and cytokines, including CRP, interleukin 6, lymphocytes, etc; symptom evaluation; virus nuclei acid tests; hospitalization time, adverse events (AE) and mortality; and TCM syndrome score. In this SR, we chose data that were more objective, consistent in the unit and completeness for analysis. Therefore, we did not analyze the TCM syndrome score or hospitalization days since the method of evaluation of TCM syndrome is different, and factors affect hospitalization time might be bias. The targeted outcomes we chose were: 1) Clinical symptoms (fever; cough; fatigue, which were measured as percentage decreased %); 2) Chest CT manifestations (the percentage of consolidations in the whole lung or improved rate %); 3) Viral nucleic conversion rates (%) and duration (days); 4) Serum interleukin-6 (pg/ml) and CRP (mg/L), and the effect estimates were re-calculated using data extracted from the qualified studies.

### 2.6 Meta-analysis

To analyze the effects of combining herbal medicines on targeted outcomes after treatment compared with baseline values, we applied Review Manager software (RevMan, Version 5.4.1, Copenhagen: The Nordic Cochrane Centre, The Cochrane Collaboration, 2014) to analyze dichotomous and continuous outcome measures extracted from the original studies. Weighted mean difference (WMD) was utilized for data measurement of continuous outcomes, while risk ratio (RR) was used for dichotomous outcomes. Statistical heterogeneity was assessed using the Chi-square test (*p* < 0.1). The I2 statistic was also calculated, and we considered *I* ^2^ > 50% to indicate significant heterogeneity across studies (Higgins et al., 2003). A random-effects model was used if significant heterogeneity was shown among trials. Otherwise, results were obtained from a fixed-effects model. Funnel plot was also used to evaluate the publication bias (Sterne et al., 2011).

## 3 Results

### 3.1 Eligible studies

**FIGURE 1:**
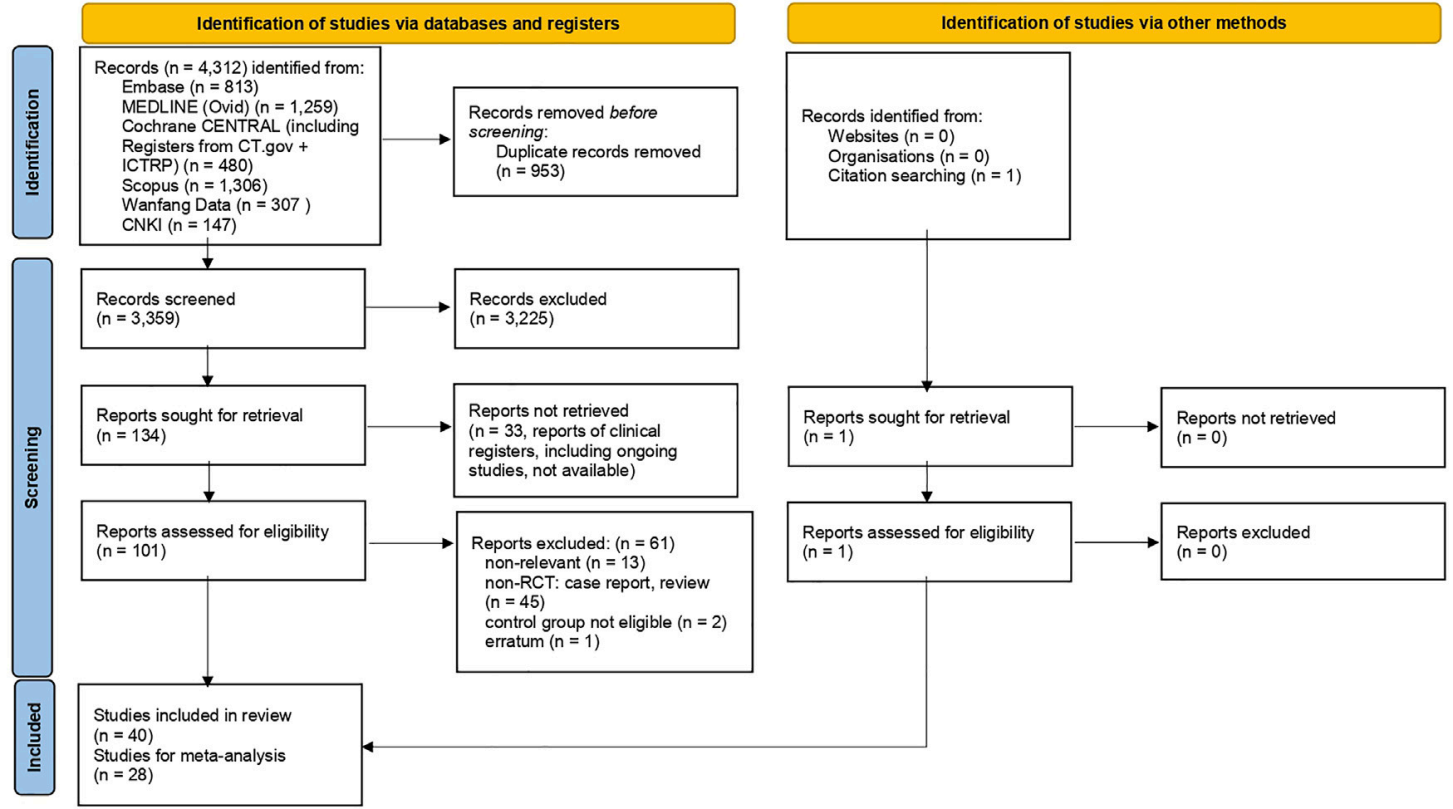
Study selection flow diagram based on the PRISMA 2020 statement.

**FIGURE 2:**
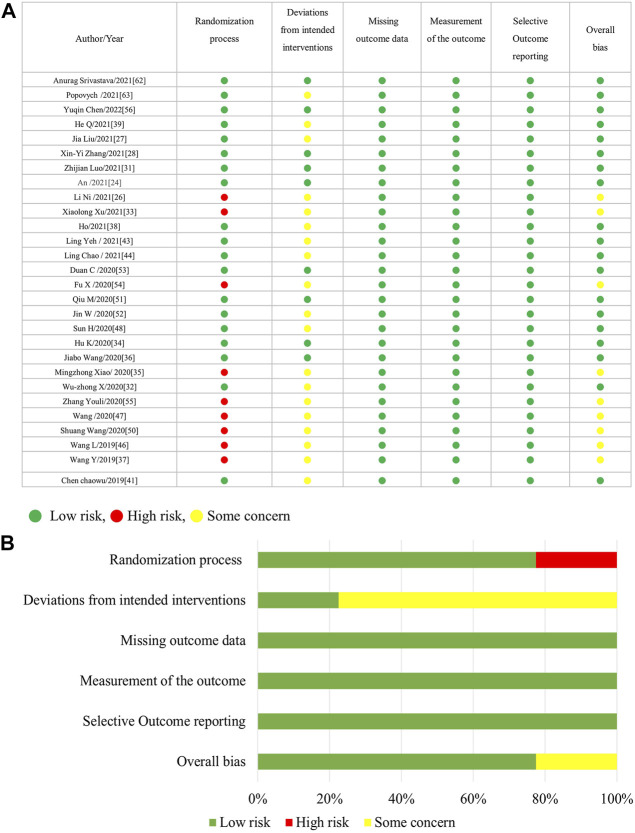
**(A)**: Risk of bias summary (ROB2). **(B)**: Risk of bias graph.

Figure 1 is the flow diagram of the literature search and screening, which complies with PRISMA guidelines (Page et al., 2021). Figure 2 demonstrates the risk of bias by applying low ROB, high ROB or some concern to each item. We also consulted the third reviewer if any disagreement occurred for risk of bias.

### 3.2 Study characteristics

In total, 40 studies that fulfilled the inclusion criteria were included for systematic review; yet 28 randomized clinical trials were eligible for meta-analysis for targeted outcome measures; Table 1 summarizes the characteristics of the 40 randomized clinical trials.

**TABLE 1:** The characteristics of the included study.

References	Sample size (intervention: Control)	Age	Type of COVID 19	Intervention	Control	Treatment days	Outcomes	Adverse report (V or no report--)
Mehrdad Karimi et al., 2021(Iran)	I:184	I:48.7	—	Persian medicine + *c*	Conventional western medicine	7	#131413	V (Gastro-intestine events)
	C:174	C:50.8					C, E,F	
Morteza Kosari et al., 2021(Iran)	I:25	I:43.5	—	Propolis plus Hyoscyamus niger L. methanolic extract	Conventional western medicine	6	C	no report
	C:25	C:41.5						
Borujerdi et al., 2021(Iran)	I:59	I:44.3	Mild to moderate	Zufa syrup	Conventional western medicine + placebo	10	C,F	no AE
	C:57	C:44.0						
Kirti S Pawar, et al., 2021 (India)	I:70	I:18–85	Mild to severe	Curcumin and piperine	Conventional western medicine + placebo	14	A, B, C, E, F	no AE
	C:70	C:25–84						
Muhammed Majeed et al., 2021 (India)	I:50	I:39.0	—	ImmuActiveTM 500 mg capsule	Placebo (same appearance)	28	C, D, E, F	no AE
	C:50	C:37.3						
Anurag Srivastava et al., 2021 (India)	I1:40	I1: 44.4	Mild to moderate	I2:Nilavembu Kudineer + allopathy treatment	Placebo + allopathy treatment	10	B, C, D,E, F	V (vomiting and diarrhea)
	I2:40	I2: 42.8		I3:Kaba Sura Kudineer				
	C:40	I3: 39.5		+ allopathy treatment				
Popovych et al., 2021 (Ukraine)	I:49	I:33.1	Mild	BNO 1030 (a standardized extract of seven medicinalplants (Imupret^®^)	Conventional western medicine	14	C, E, F	no AE
	C:47	C:3.6						
Yuqin Chen et al., 2022(China)	I:64	I:54.2	—	Bufei Huoxue capsules	Placebo	90	A, C, F, G	V (Abnormal liver function)
	C:65	C:52.5						
He Q, et al., 2021(China)	I:36	—	Mild	Buzhong Yiqi decoction + *c*	Conventional western medicine	10	A, B, C,D,F	V (1 with arrthymia)
	C:35							
Jia Liu et al., 2021(China)	I:99	I:56.0	Mild to severe stages	Huashi Baidu granule + *c*	Conventional western medicine	14	A, B, C, D, F	V (diarrhea)
	C:96	C:56.5						
Xin-Yi Zhang et al., 2021(China)	I:65	I:44.3	Mild to moderate	Xiyanping injection + *c*	Conventional western medicine	14	C,D,F	V (mild)
	C:65	C:48.3						
Zhijian Luo et al., 2021(China	I:29	I:60.3	Severe	Xuebijing	Conventional western medicine	14	B,C,E,F	V (no AI)
	C:28	C:56.4						
Shuang Zhou et al., 2021(China)	I:57	66 (median)	Severe/Critical	Standard care (Table 1)+shenhuang granule	Conventional western medicine	14	B, F	**V (**Increased neutrophil)
	C:54							
An et al., 2021(China)	I:92	I:50.2	—	Jinhua Qinggan granules	Conventional western medicine	14	C,D, F	no AE
	C:31	C:44.7						
Li Ni et al., 2021(China)	I:56/61/59	I:54/56/53	—	Shuanghuanglian oral liquids	Conventional western medicine	14	A, C, D, F	No serious adverse events occurred
	C:59	C:51						
Xiaolong Xu et al., 2021(China)	I:77	I:49.1	—	Reduning injection	Conventional western medicine	14	C, D, E, F	V
	C:80	C:50.4						
Congcong Zeng et al., 2021(China)	I:30	I:50.7	Mild to moderate	Maxingshigan-Weijing Decoction	Conventional western medicine	14	B, C, D, E, G, F	no AE
	C:29	C:53.3						
Chen Zhao et al., 2021(China)	I:358	I:52.0	Mild	Huashibaidu Granule	Conventional treatment	7	C, E, F	V (Mild diarrhea)
	C:384	C:50						
Ping Xianghua et al., 2021(China)	I:30	I:40.8	Mild to severe	Jiawei Yupingfeng powder	Conventional western medicine	14	A, B, C, D, E, F	V (vomiting, chest distress diarrhea)
	C:24	C:41.2						
Ho et al., 2021(China)	I:34	I:15–80	Moderate to severe	Shengmai Powder	Conventional western medicine	7	A, B, D, G	no report
	C:30	C:15–80						
Zhang Dan et al., 2021(China)	I:240	—	High risk	Fuzheng Gubiao Fangggan Decoction	Conventional western medicine	14	C	no report
	C:240							
Ling Yeh et al., 2021(China)	I:50	I:43.3	Ordinary	Modified Shengjiang Powder	Conventional western medicine treatment	6	B, C, F	no AE
	C:50	C:42.6						
Ling Chao et al., 2021(China)	I:51	I:46.8	Ordinary	Antivirus No. 1 + *c*	Conventional western medicine treatment	9	A, B, C, G	no report
	C:45	C:45.1						
Liu et al., 2021(China)	I:15	I:41.6	Mild to moderate	Jiawei Sang Ju drink	Conventional western medicine	10	A, B, C, D, F	V (no AI)
	C:15	C:44.5						
Duan C et al., 2020 (China)	I:82	I:52.0	Mild	jinhua qinggan granules	Conventional western medicine	5	C, G,F	V (diarrhea)
	C:41	C:50.3						
Fu X, et al., 2020(China)	I:37	I:45.3	Ordinary	Toujie Quwen granule	Conventional western medicine	15	B, C, F	no AE
	C:36	C:44.7						
Qiu M et al., 2020(China)	I:25	I: 53.4	Ordinary	Maxing Xuanfei Jiedu decoction	Conventional western medicine	10	A, C, G	no report
	C:25	C:51.3						
Jin W et al., 2020(China)	I:20; C:18	I:43.6	Ordinary	Compound Yin Chai granule + Qingqiao detoxification granule	Routine western medicine	21	A, B, C, E, F	no report
		C:41.3						
Sun H et al., 2020(China)	I:32	I:45.4	Mild, Ordinary	Lianhua Qingke granule + C	Antiviral medications	14	A, C, F	no AE
	C:25	C:42.0						
Hu K et al., 2021 (China)	142:142	I:50.4; C:50.8	Ordinary	Lianhua Qingwen capsules (1.4 g,tid)+C	Antiviral medications	14	A, C, D, F	V (liver function)
Jiabo Wang et al., 2020(China)	I:24	I:46.8	—	Keguan-1 (ARDS-suppressing drug)+C	Antiviral drugs	14	A, D, F	V (diarrhea, anorexia, vomiting)
	; C:23	C:51.4						
Mingzhong Xiao et al., 2020(China)	I:119 (58/61)	I:52.6/56.7	—	1)Huoxiang Zhengqi dropping pills and Lianhua Qingwen granules or 2) Linahua granules	Antiviral drugs	14	C, F	no AE
	; C:63	C:53.9			Anti-infective drug			
Wu-zhong **Xiong** et al., 2020(China)	I:22	I:57.1; C:62.4	Mild to ordinary	Xuanfei Baidu decoction + *c*	Conventional western medicine	7	B, C, F	no AE
	C:20							
Zhang Youli et al., 2020(China)	I:80	I:53.4	Ordinary	Jinyinhua Oral Liquid + C	Conventional western medicine	10	A, C, D	V (diarrhea)
	C:40	C:52.0						
Wang LQ et al., 2020(China)	I:58	I:66	Mild to severe	Gegen Qinlian pill	Routine treatment	—	A,B, C, D, F	no AE
	C:60	C:57.5						
Mou et al., 2020(China)	I:37	I:47.4	Mild to severe	Maxing Shigan Sanren Decoction	Conventional treatment	—	C	no report
	C:37	C:42.2						
Shuang Wang et al., 2020(China)	I:45	I:43.8	Ordinary	Tablets Combined with Sanren Decoction	Western medicine	15	B, C	no report
	C:45	C:42.6						
Wang L et al., 2020(China)	I:40	—	Ordinary	Shengmai powder + Shenling Baizhu powder	Conventional western medicine	—	A, B, C, D, G, F	no AE
	C:40							
Wang Y et al., 2021(China)	I:70; C:70	I:48.0	Ordinary	Qingfei Paidu decoction + *c*	Conventional western medicine	10	B, E, F, G	V (fatigue)
		C:49.4						
Chen chaowu, et al., 2021(China)	I:28	I:49.5	Mild	Lianhua Qingwen capsule + *c*	Conventional western medicine	—	B, C, D, F	V (vomiting, diarrhea, liver)
	C:29	C:50.2						

Outcomes: A: Chest CT, or other imaging; B: blood test and cytokine; C: symptom evaluation; D: virus nuclei acid tests; E: hospitalization time; F: AE, and mortality; G: TCM, syndrome score.

Among these 40 randomized clinical trials, 33 trials were conducted in China with Chinese herbal medicine; of these, 13 were published in English (Wang J. B. et al., 2020; Xiong W. Z. et al., 2020; Xiao et al., 2020; An et al., 2021; Zhao C. et al., 2021; Liu J. et al., 2021; Xu X. et al., 2021; Zhang X. Y. et al., 2021; Hu et al., 2021; Luo et al., 2021; Ni et al., 2021; Zeng et al., 2021; Zhou et al., 2021), while 20 were published in Chinese (Jin W. et al., 2020; Wang et al., 2020b; Wang et al., 2020c; Duan et al., 2020; Wang S. et al., 2020; Fu et al., 2020; Mou et al., 2020; Qiu et al., 2020; Sun et al., 2020; Zhang et al., 2020; Chen C. et al., 2021; Liu A. et al., 2021; Chen Y. et al., 2021; Zhao J. et al., 2021; Wang Y. et al., 2021; He et al., 2021; He and Zhang, 2021; Ping et al., 2021; Ye et al., 2021; Zhang, 2021). In addition, 3 studies were conducted in Iran (Karimi et al., 2021; Kosari et al., 2021; Borujerdi et al., 2022), 3 in India (Majeed et al., 2021; Pawar et al., 2021; Srivastava et al., 2021) and 1 in Ukraine (Popovych et al., 2021). Twelve randomized clinical trials (30%) were multi-center trials whereas others were conducted in a single site. In total, 5,417 study participants were included in this systematic review, with sample sizes ranging from 15 to 384. Treatment duration ranged from 6 to 90 days.

### 3.3 Assessment of methodological quality

Figure 2 shows that 33% of the included trials (13/40 randomized clinical trials) did not appropriately address the process of randomization, and more than 75% (31/40 trials) have the deviations from intended interventions. Regarding selective outcome reporting and incomplete outcome measures data, all included trials were within low risk; however, some trials were within unclear risk of allocation concealment. Generally speaking, the included trials were of medium quality, while details of randomization and blinding were the most frequent problems.

#### 3.3.1 Grading of recommendations assessment development evaluation assessment

Table 2 summarizes the evidence certainty of outcomes. Considering that more than half of the enrolled randomized clinical trials were rated as some-concerned of bias (RoB), we downgraded the evidence certainty in the domain of study limitation. The domain of inconsistency was downgraded because of the varied heterogeneity in outcomes as how to evaluate the severity of cough and fever. Publication bias was not considered based on no asymmetry in funnel plots.

**TABLE 2:** Grade evidence Profile.

Certainty assessment							Risk difference 95% CI
Outcomes (no of studies)	Study limitation	Inconsistency	Indirectness	Imprecision	Publication bias	Overall certainty of evidence	
Fever (12 randomized clinical trials)	No serious limitation	No serious inconsistency	No Serious indirectness	No serious	Undetected	Moderate	1.09 (1.1.19)
						⊕⊕○○	
Cough (13)	Serious	Serious inconsistency	Serious indirectness	Serious	Undetected	Low	1.43 (1.21, 1.71)
						⊕○○○	
Fatigue (11)	Serious	Serious inconsistency	Serious indirectness	Serious	Serious	Low	1.21 (1.10.1.33)
						⊕○○○	
Chest CT (16)	No serious	No serious	No Serious indirectness	No serious	Undetected	High	1.26 (1.19.1.34)
						○⊕⊕⊕	
Virus conversion time (10)	No serious	No serious	No Serious indirectness	No serious	Undetected	High	1.22 (1.06.1.40)
						○⊕⊕⊕	
Virus conversion rate (5)	No serious	No serious	No Serious indirectness	No serious	Undetected	High	−3.72 (−6.05, −1.40)
						○⊕⊕⊕	
Interleukin -6 (4)	No serious	No serious	No Serious indirectness	No serious	Undetected	High	1.97 (−0.72.4.66)
						○⊕⊕⊕	
CRP (10)	No serious	No serious	No Serious indirectness	No serious	Undetected	High	−7.92 (−11.3,−4.53)
						○⊕⊕⊕	

GRADE: grading of recommendations assessment, Development, and Evaluation; RCT: randomized clinical trials; CI: confidence interval.

### 3.4 Publication bias

**FIGURE 3:**
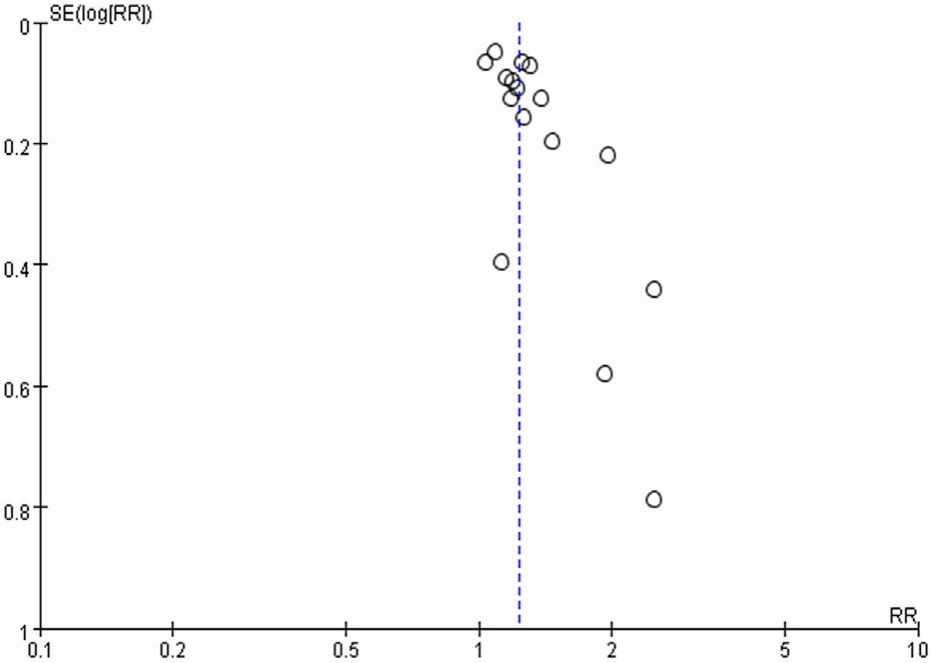
Funnel plots for included studies.

The funnel plot was used to explore potential publication bias (Figure 3). The funnel plot is symmetrical, indicating no obvious deviation and that publication bias is unlikely. Thirty-four trials (85%) reported “No” adverse events (AE), indicating little bias in this systematic review.

### 3.5 Main traditional chinese medicine and herbal components included in data analysis

This meta-analysis includes not only TCM but also HM, and 90% of the included trials used TCM formulas or granules. The complexity of TCM or HM components is evident; the most popular components are ***honeysuckle- and forsythia-*** based (which is a concept of ***couplet medicines*** in TCM theory), such as Jinhua qinggan granules (An et al., 2021), Jinyinhua liquid (Duan et al., 2020; Zhang et al., 2020), Toujie Quwen Granules, and Lianhua qingwen capsules (Duan et al., 2020; Yu et al., 2020; Chen C. et al., 2021; Hu et al., 2021). Another popular couplet medicines applied in these studies is ***ephedra sinica-*** and ***almond***-based, such as Huashi Baidu granules (Zhao C. et al., 2021), Qingfei Paidu decoction (Wang Y. et al., 2021), Maxingshigan-Weijing decoction, Maxing Xuanfei Jiedu decoction (Qiu et al., 2020), Lianhua qingwen capsules (Chen et al., 2020; Yu et al., 2020; Chen C. et al., 2021; Hu et al., 2021) and Guangwenyilun. Other herbs are applied in India, Iran and Ukraine, including curcuminoids (Pawar et al., 2021), Propolis (Kosari et al., 2021), Rheum palmatum (Karimi et al., 2021) and polyherbal formulas (Borujerdi et al., 2022). The components of herbal medicine used in each trial are listed in Supplementary Appendix A2.

### 3.6 Outcomes and efficacy assessment

#### 3.6.1 Effects of combined -herbal medicines on COVID-19 clinical symptoms (fever; cough; fatigue)

**FIGURE 4:**
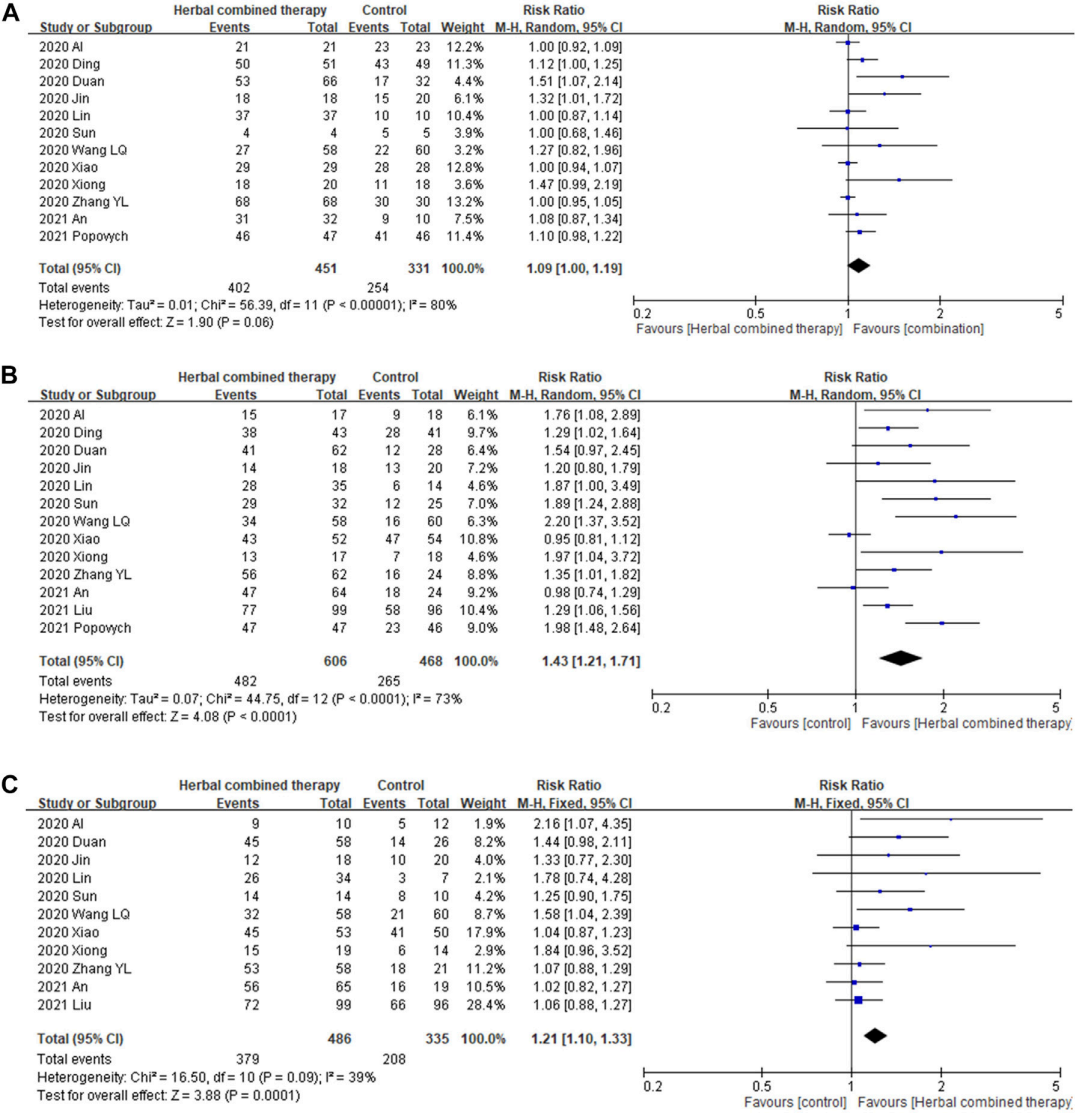
Forrest plot of the effects of HM combined therapy in symptom relief. **(A)**: comparison of HM combined therapy in fever reduction. **(B)**: Comparison of HM combined therapy in cough reduction. **(C)**: Comparison of HM combined therapy in fatigue reduction.

Significant between-study heterogeneity was observed in the effects of combined herbal medicines on COVID-19-related fever and cough (I2 = 80%, and 73%, respectively), but no significant between-study heterogeneity was noted in the effects of combined herbal medicines on fatigue (I2 = 39%). For the 12 trials that reported data on fever reduction cases, no significant differences were observed in subjects treated with combined HM and CWM (1.09 95% CI:1.00, 1.19, *p =* 0.06; Figure 4A) as compared with control intervention. In the 13 trials that reported data on cough reduction cases, significant improvement was observed in subjects treated with add-on herbs (1.43 95% CI:1.21, 1.71, *p =* 0.0001; Figure 4B) as compared with control subjects. In the 11 trials that reported data on fatigue reduction cases, significant differences in patient benefits were found when they were treated with combined HM and CWM (1.21 95% CI:1.10, 1.33, *p =* 0.0001; Figure 4C).

#### 3.6.2 Effects of combined -herbal medicines on chest CT manifestations in COVID-19 patients

**FIGURE 5:**
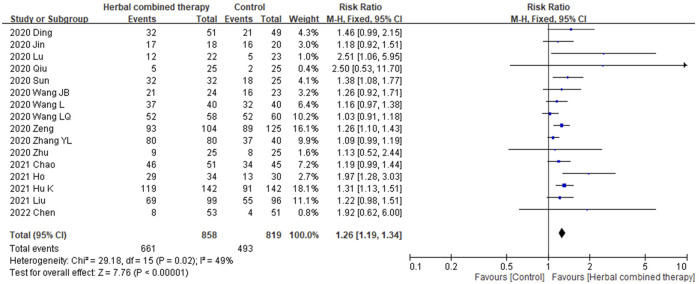
Forrest plot of the effects of HM combined therapy in chest CT improvement.

No significant between-study heterogeneity in chest CT manifestations were noted between studies in terms of combining HM and CWM (I2 = 49%). As for the 16 trials that reported chest CT data, significant improvement in CT images was observed in subjects receiving add-on herbal medicine (1.26 95% CI:1.19, 1.34, *P* ≤ 0.00001; Figure 5) as compared with those of control subjects. We provide additional random model effect in Supplementary Appendix A3.

#### 3.6.3 Effects of combined -herbal medicines on viral nucleic test, and viral negative conversion time (days)

**FIGURE 6:**
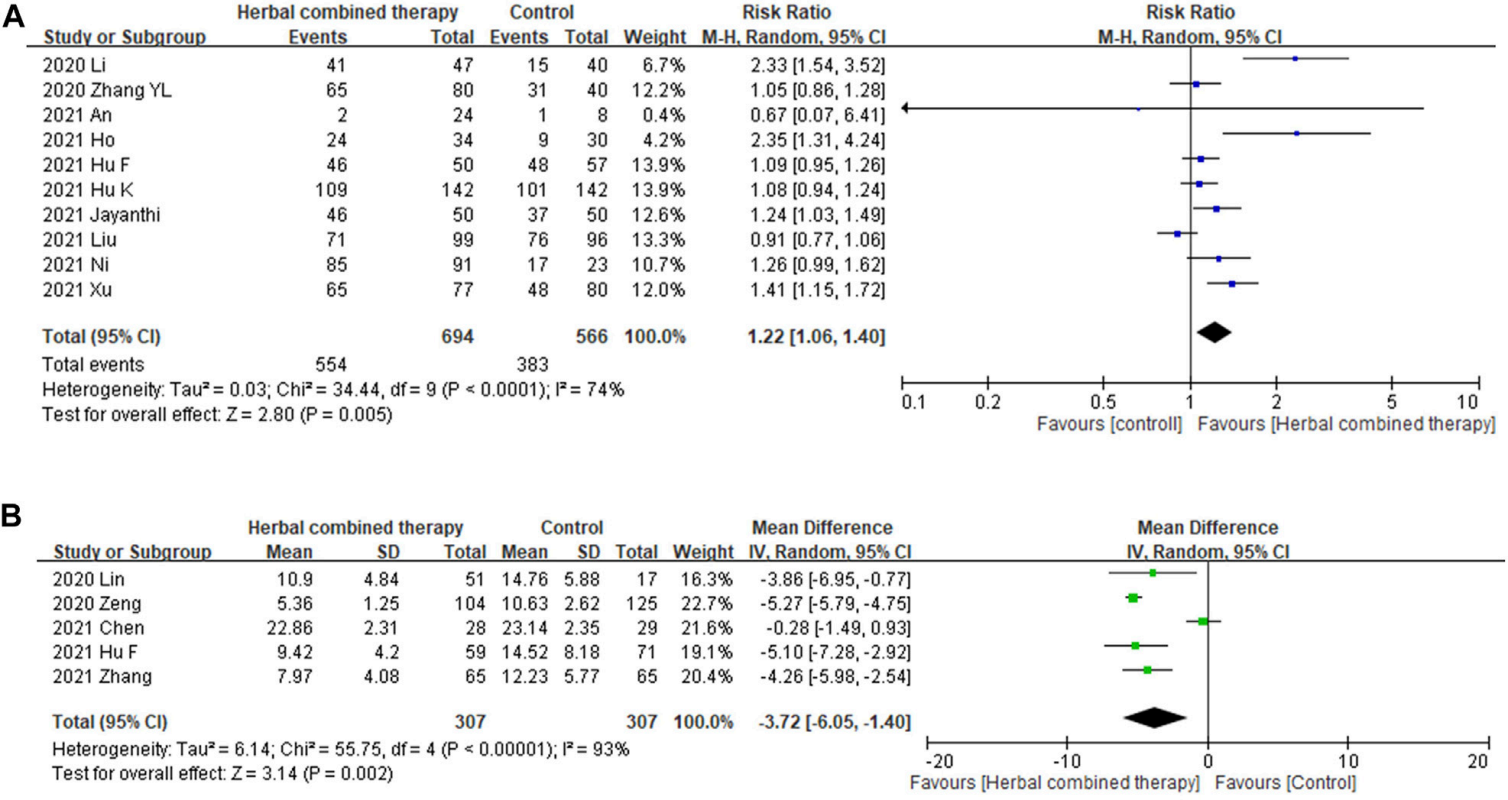
**(A)**: Forrest plot of the effects of HM combined therapy in viral conversion rate. **(B)**: Forrest plot of the effects of HM combined therapy in viral conversion time (day).

Significant between-study heterogeneity was observed in the effects of alternative medicines on viral conversion rate and viral conversion time (*I* ^*2*^ = 74%, and 93%, respectively). For the 10 trials that reported data on viral negative conversion rates, more significant improvements were observed in subjects whose treatment included with add-on herbs (1.22 95% CI:1.06, 1.40, *p =* 0.005; Figure 6A). In the 5 trials that reported data on viral negative conversion time (days), significant improvement was noted in subjects whose treatment included combined herbs (−3.72 95% CI: −6.05, −1.40, *p =* 0.002; Figure 6B) as compared with CWM alone.

#### 3.6.4 Effects of combined -herbal medicines on serum interleukin-6 (IL-6), and C reactive protein

**FIGURE 7:**
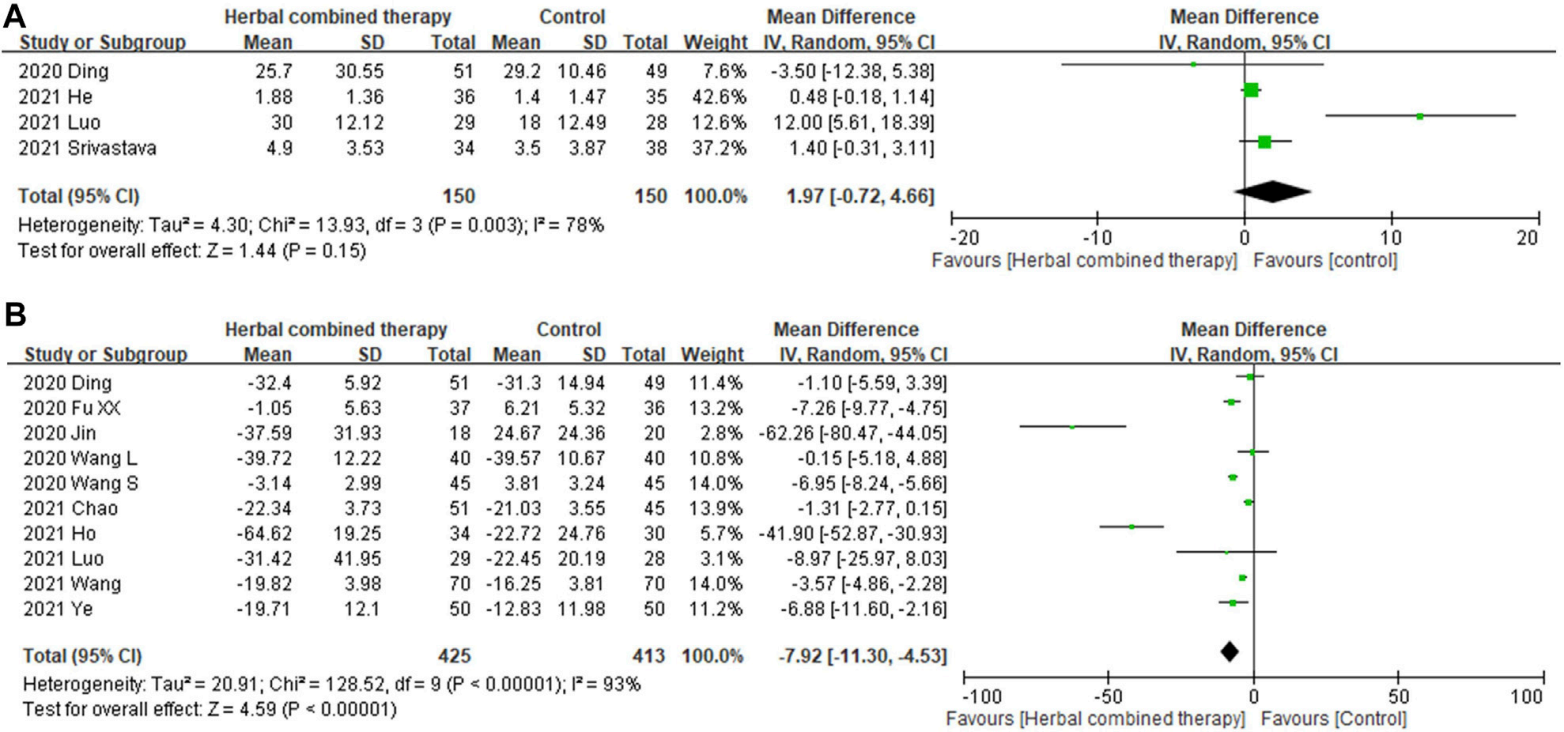
**(A)**: Forrest plot of the effects of HM combined therapy in interleukin-6 (ng/ml) in COVID19 patient. **(B)**: Forrest plot of the effects of HM combined therapy in C-reactive protein (ng/ml)in COVID19 patient.

Significant between-study heterogeneity was observed in the effects of alternative medicines on serum interleukin-6 level and CRP level (*I* ^*2*^ = 78%, and 93%, respectively). In 4 trials that reported data on serum interleukin-6 level, no significant differences were observed in subjects treated with add-on herbs (1.97 95% CI: −0.72, 4.66, *p =* 0.15; Figure 7A). Regarding serum CRP levels, significant decreases were noted in the analysis of 10 enrolled trials that treated with combined HM (−7.92 95% CI: −11.30, −4.53, *p =* 0.000; Figure 7B) as compared with control subjects.

#### 3.6.5 Safety concern

No serious adverse events were noted in all included randomized clinical trials, except few trials mentioned that participants might have some nausea or GI upset which not directly related to the herbal formula. Accordingly, no quantitative analysis was applied targeted the safety concern or adverse events.

## 4 Discussion

To our knowledge, this meta-analysis has the advantages of recruiting 2-years trials, and that outcomes selection was not only confined to subjective constitution scores or subjective symptom improvement but also included the objective measurement of changes in CT images, virus conversion rate and time and cytokines such as IL6, as well as changes in CRP levels. Except for IL6 changes, the results favored HM combined therapy by its add-on effect, which shortened virus conversion time and rate and improved clinical symptoms and CRP better than CWM alone. The mechanism of HM is hard to confirm since the components of these formulas are complicated, ranging from a single herb to more than ten herbs added into the treatment regimen in these trials.

Furthermore, the dosage and the course of treatment can always be adjusted according to the patient’s condition, for which sensitivity analysis shows increased heterogeneity. One study even tried to identify the most frequently-used herb associated with this issue (Xiong X. et al., 2020), but as each herb’s proportion varies between different formulas, and the use of HM or TCM emphasizes the synergistic effect between multiple herbs in individual formulas, it remains difficult to identify a specific single herb as a potential drug for COVID-19 treatment. However, Indian trials applied Zingiber officinale-based (Srivastava et al., 2021) or curcumin-based (Pawar et al., 2021) polyherbal formulas, which are also popularly used in Asian countries. Several TCM studies focused on “Lian-Hua Qing-Wen granules” (Hu et al., 2020; Shi M. et al., 2021) and Qingfei Paidu decoction (Li X. et al., 2021; Li Y. et al., 2021; Wang Q. et al., 2021; Wu et al., 2022), which are recognized as Chinese patent medicines and are now available in western countries. In the real world, HM emphasizes the synergistic effect based on holistic theory, which appeals to people who prefer natural products. In the academic field, some scholars have applied modern pharmacologic methods in analyzing TCM formulas and noted that they have similar mechanisms, including the regulation of apoptosis and immune response (Shi et al., 2021c), and anti-inflammatory effects, which tend to benefit lung function in patients with COVID-19 (Yan et al., 2020; Zhang F. et al., 2021). For example, a deep study proposed that “Rheum palmatum L (Da Huang),” a common component of these formulas, can directly block the viral life cycle (Shi et al., 2020), and inhibit viral transcription and replication (Galasiti Kankanamalage et al., 2018). The meta-analysis noted that combining HM therapy decreases CRP significantly, but does not decrease Il-6, indicating that HM helps to reduce the inflammatory status through an uncertain pathway. In a previous study, the CRP level correlated with the severity of lung involvement and prognosis (Beydogan and Yuruk Atasoy, 2021). In this context, it makes sense that many Chinese herbal medicines utilized in these trials targeted the lung meridian and anti-inflammatory effects are especially valuable. Similar studies investigated the potential herbs by utilizing information medicine and precision medicine (Balkrishna et al., 2021; Dzobo et al., 2021). While the present study has confirmed the efficacy of HM, further studies are needed to connect the potential drugs with precision medicine.

Additionally, some of the outcomes in the enrolled trials such as TCM syndrome, hospitalization days, or adverse events were not analyzed is due to the evaluation methods of TCM syndrome are not consistent or well addressed in original manuscripts. We didn’t analyze the hospitalization days owing to it was probably affected by some other medical, social, and medical rules problems which not directly related to COVID-19 infection. Furthermore, most studies address the adverse events in description without quantitative comparison; therefore, some outcomes were mentioned in the enrolled trials yet cannot be analyzed. Generally speaking, since the COVID-19 pandemic started in 2019, and researchers are still very much at a groping stage. Some consensuses are under modified during this period; thus, we only chose the presentive clinical symptom (fever, cough, fatigue), image improvements, and quantitative outcomes as cytokine change and reliable virus conversion times and rate for analysis. Further detailed analysis towards other items could be considered depend on how scholars treat the COVID related issues.

Regarding the basic mechanism about these herbs or potential of the plants’ extracts, more and more research focus on the network pharmacology analysis and cross-talks between signaling pathways (Qi et al., 2022). Take some formulas, which have been included in this meta-analysis, Lian Hua Qing Wen has been proved with regulating angiotensin converting enzyme 2 (ACE2) expression-disorder-caused symptoms and relieve the cytokine storm (Zheng et al., 2020). Some study revealed that acacetin, wogonin, and isorhamnetin were the main active ingredients in Qing Fei Pai Du decoction, (Li et al., 2022); while the target network model noted that some compounds such as licochalcone B acted on multiple targets, and multiple components interacted with the same target such as GPR35, reflecting the synergistic mechanism of Chinese medicine (Xu F. et al., 2021). Another special agent which not included in this trial is a potential mucosal topical agent “Ankaferd hemostat (ABS)”, which was approved with effect of antagonizing proteinase-activated receptors (PARs), mainly PAR-1. By activation of the PAR-1, mediators and hormones impact on the hemostasis, endothelial activation, alveolar epithelial cells and mucosal inflammatory responses which are the essentials of the COVID-19. The mucosal problem is an issue which has been ignored for most study focused on oral form and systematic treatment (Beyazit et al., 2020).

The basic research of herbs confronts the problems that in-depth studies are not available to precisely define bioactive compounds of plant origin and their mechanism of action as many cross-talks between target pathway network and protein-protein interaction (PPI) network (Wang L. et al., 2021); this underlines the need for studying the synthetic molecules to circumvent the viral load in the host system (Prasad et al., 2020). Additional trials or basic research may be designed on the hypothetically established plant extracts as add-on herbal medicines in the COVID-19 treatment field and there is a lot space for exploration.

Lastly, although some between -study heterogenicity was noted; we didn’t perform additional heterogeneity analysis as no more than 10 trials among these indicators are included and we still need to hold a conservative view point towards the results. Furthermore, no un-tolerable side effects were reported by 80% of the studies in the present meta-analysis. Among the enrolled trials, the treatment goals primarily targeted respiratory distress and symptoms. In the present review, the most common AEs were diarrhea or other gastrointestinal upset, which may also be associated with anti-inflammatory effects of the specific herbal medicines applied for treating COVID-19 (Luo et al., 2020). Therefore, the side effects were similar to those of antibiotics or associated with combining the herbs with the use of western medicines (anti-viral, antimicrobial agent); nevertheless, the events were mild and tolerable, we didn’t analyze since there were no quantitative report from the enrolled trials. However, another study advocated cautious use of HM in patients with prominent gastrointestinal symptoms (Shi et al., 2021b). We suggest that in the process of HM research, drug-drug interactions remain a concern in mainstream medicine, which prompts us to explore new drugs, not only those confined to herbal medicine. Therefore, the best way is to record AEs carefully, and the present review has revealed that HM is safe and the side effects are easily manageable. Accordingly, in this meta-analysis, patients with mild to moderate COVID-19 infection who received conventional therapy combined with HM benefited more than those receiving only CWM. From an epidemiological view, because COVID-19 is caused by rapid viral emission, shortening the disease is meaningful toward decreasing the spread of the pandemic and lowering the medical and economic burden (Pak et al., 2020; Zhang et al., 2022). Since the global population has paid a heavy price for this pandemic, it is worthwhile to make the best use of herbal medicines in treating infected patients worldwide.

### 4.1 Limitations

The present review has several limitations. First, many included trials lack details of methodology such as the randomization process, allocation or blinding. Secondly, the composition of herbal formulas varied considerably between studies and some even had overlapping components. Thirdly, most of the trials were conducted in a single center, which limits generalizability of results to other populations and may also compromise comparisons with multicenter studies. Lastly, more consensus about the reliable endpoints were in need to be reached about herbal medicine related study before we can make any further precise analysis and conclusion. As COVID-19 is an emergent infective disease, double blinding is difficult, yet in further study, more consistent herbal formulas and solid methodologies are required to make a powerful conclusion. We also expect more and more herbal medicine related COVID-19 research will published, and we might extract some studies which are qualified for sub-group analysis and provide valuable information for future scholars who has interests in mechanism exploration; and this is our preliminary meta-analysis on this issue.

## 5 Conclusion

This systematic review has demonstrated that herbal medicine is a viable complementary therapy for COVID-19 and the application of herbal medicine to COVID-19 patients in certain circumstances is recommended. It has the benefit of mitigating clinical symptoms (fever, cough, fatigue) and shortening the disease duration; yet for the complicated components in varied formulas, of which herb exert its effect need to be further investigated and clarified. The definitive herb or mechanism remains uncertain and related studies are ongoing. The merge of existing studies and further identify potential herb for following studies with scientific method is in emergent. Further large and rigorous multicenter trials or basic research integrating informative and precision medicine are warranted in order to clarify role of add-on herbal medicine in the COVID-19 regimen.

## Data Availability

The original contributions presented in the study are included in the article/Supplementary Material, further inquiries can be directed to the corresponding author.

## Abbreviations

HM, Herbal medicine; CWM, conventional western medicine; WHO, World Health Organization; RoB 2, Risk of Bias version 2; RCTs, randomized controlled trials; AE, adverse events; RR, risk ratio; ACE2, angiotensin converting enzyme2.
