# Supplementary material for: Therapeutic effects of herbal-medicine combined therapy for COVID-19: A systematic review and meta-analysis of randomized controlled trials
Source: Front Pharmacol. 2022 Sep 1;13:950012. doi: 10.3389/fphar.2022.950012 (PMC9475194; doi:10.3389/fphar.2022.950012)
Supplement: Supplementary file 1 [file DataSheet2.PDF]

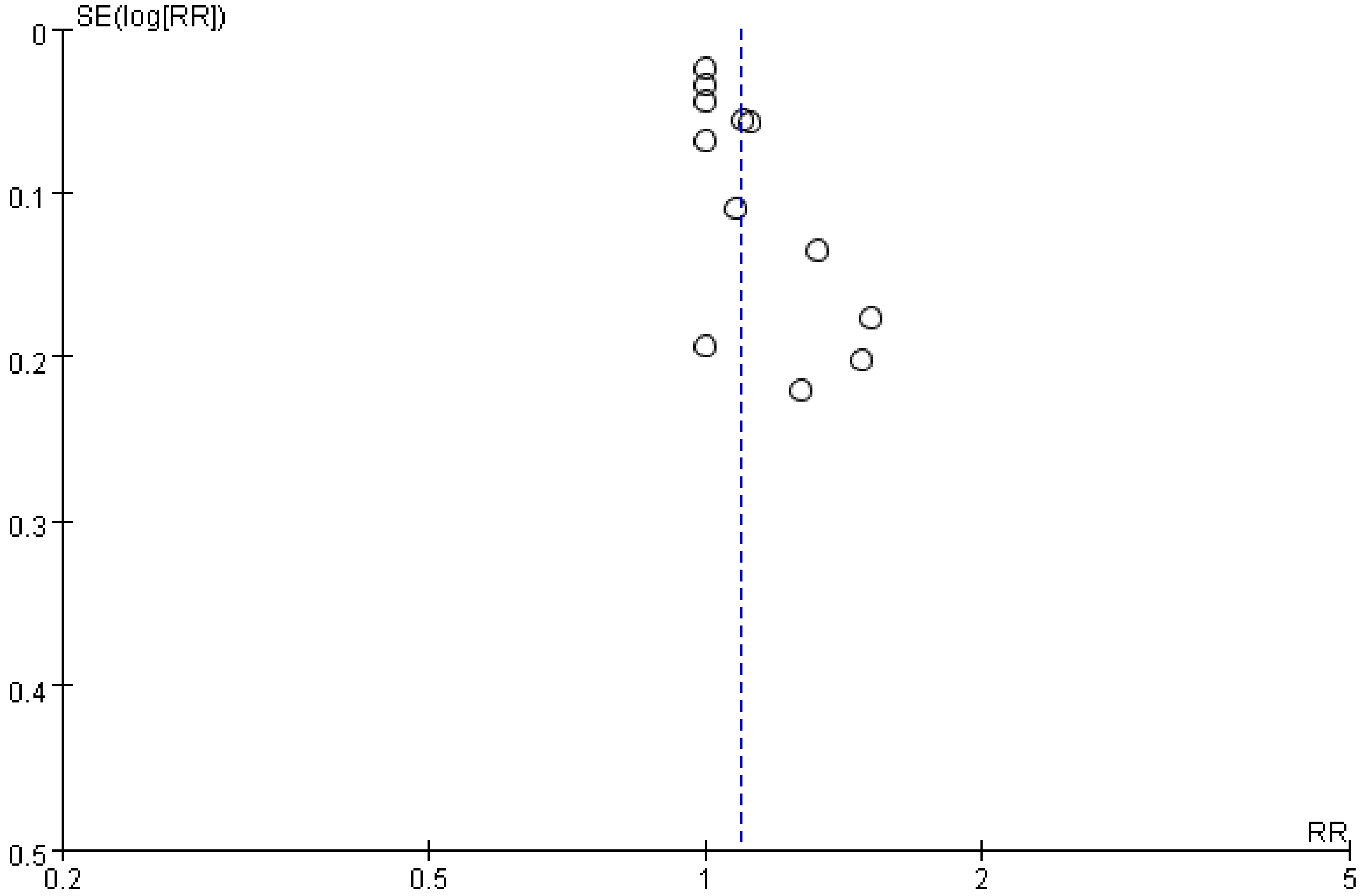

Figure: funnel plots for included studies for fever

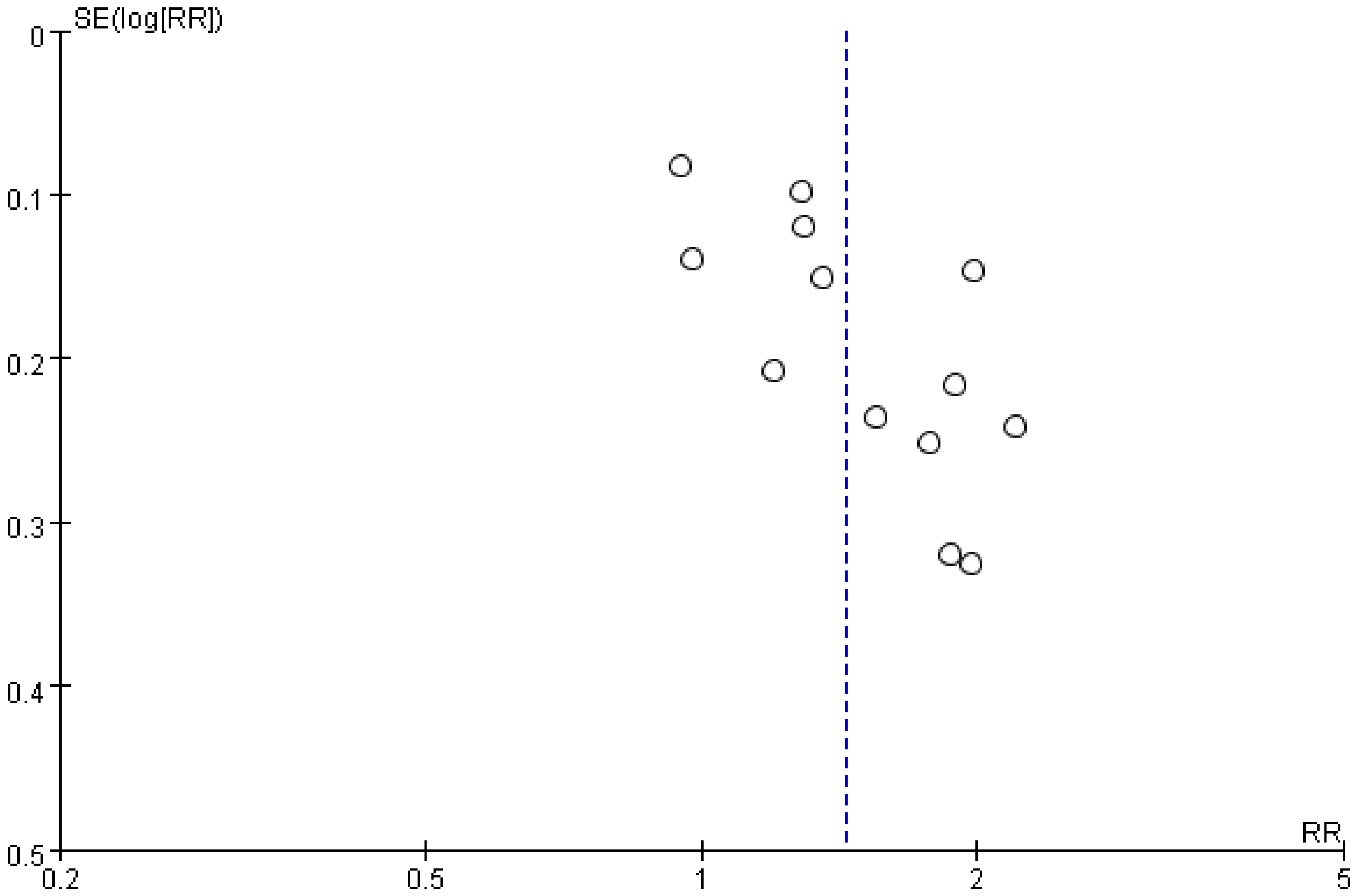

Figure: funnel plots for included studies for cough

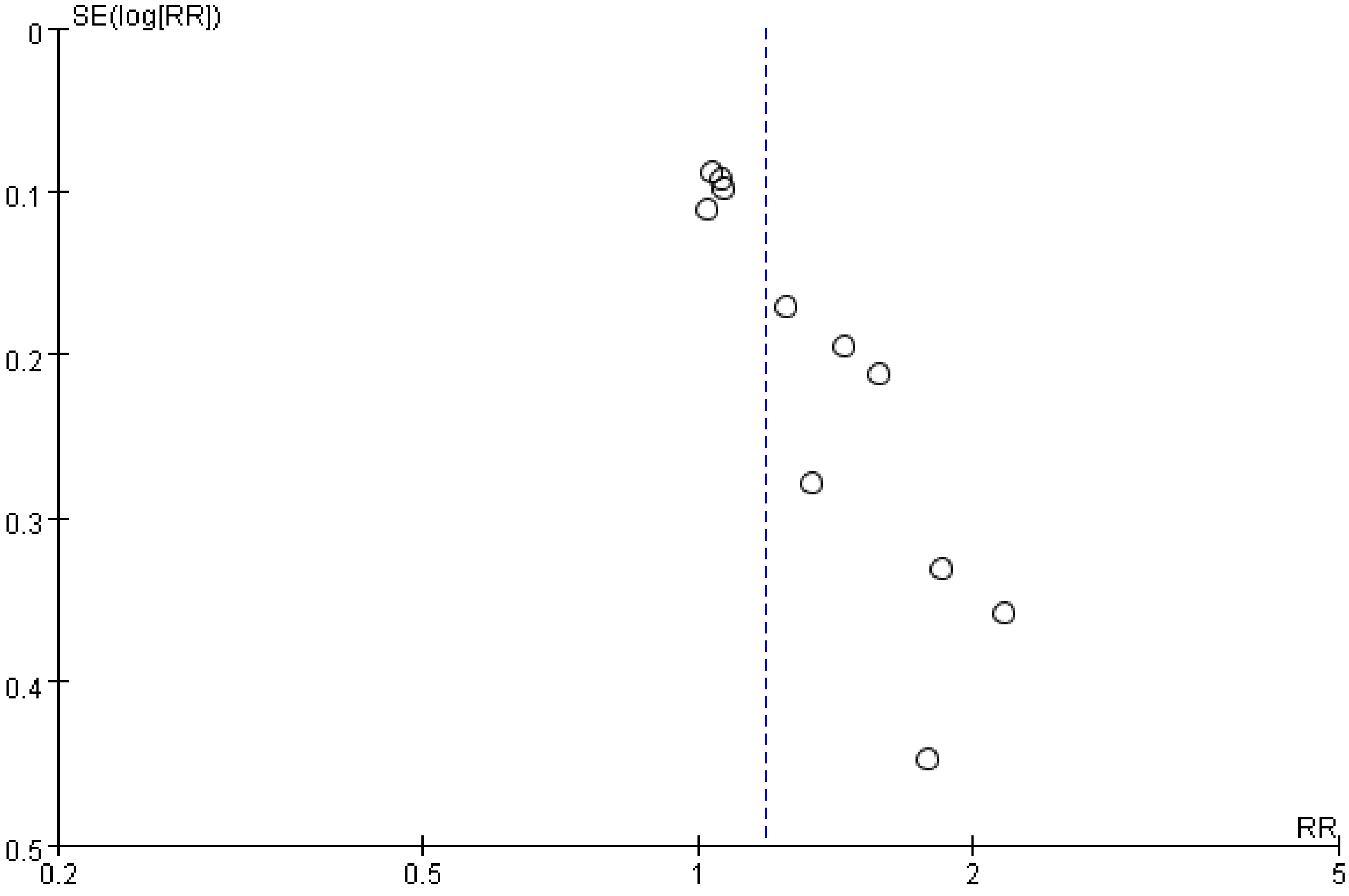

Figure: funnel plots for included studies for fatigue
