# Supplementary material for: Therapeutic effects of herbal-medicine combined therapy for COVID-19: A systematic review and meta-analysis of randomized controlled trials
Source: Front Pharmacol. 2022 Sep 1;13:950012. doi: 10.3389/fphar.2022.950012 (PMC9475194; doi:10.3389/fphar.2022.950012)
Supplement: Supplementary file 2 [file Table1.DOCX]

**Appendix 1：Search Strategy（Trational Medicine for COVID-19）**

| **Database** | **#** | **Search Syntax** | **Results** |
| --- | --- | --- | --- |
| **Embase** | 1 | (COVID OR "HCoV-19" OR "new coronavirus*" OR "novel coronavirus*" OR "SARS coronavirus* 2" OR "SARS-CoV-2" OR "SARS-CoV2" OR "SARS-CoV-II" OR "SARS 2" OR SARS2 OR nCoV OR nCOVID OR "severe acute respiratory syndrome coronavirus 2" OR "severe acute respiratory syndrome 2" OR "wuhan seafood market pneumonia virus*" OR ((wuhan OR chinese OR china) NEAR/3 coronavirus*)):ti,ab,kw |  |
|  | 2 | "coronavirus disease 2019"/exp or "Severe acute respiratory syndrome coronavirus 2"/exp |  |
|  | 3 | (coronavirus* OR COV OR sars OR "severe acute respiratory syndrome"):ti,ab,kw AND [2019-3000]/py |  |
|  | 4 | ("coronaviridae infection"/de OR "coronavirus infection"/de OR "severe acute respiratory syndrome"/de OR "coronaviridae"/de OR "coronavirinae"/de) AND [2019-3000]/py |  |
|  | 5 | ((traditional NEAR/4 medic*) OR TCM OR CAM OR herb* OR phytotherap* OR phytomedicine OR "chung i" OR "zhong yi" **OR** ((chinese OR orienta* OR kampo OR botanic* OR phyto* OR plant*) NEAR/6 (drug* OR medic* OR formula* OR remed* OR preparation* OR capsule* OR pellet* OR solution* OR pill* OR powder* OR granule* OR granula* OR extract* OR product* OR agent* OR substance* OR material* OR traditional OR phyto* OR plant* OR therap* OR treatment* OR intervention* OR supplement*)**) OR (**(complementary OR alternative) NEAR/6 (drug* OR medic* OR formula* OR remed* OR preparation* OR therap* OR treamment* OR intervention*)**) OR** fang OR tang OR wan OR san OR dan OR decoction* OR NRICM101 OR ChingGuan OR "Ching Guan" OR JinHuaQingGan OR "JinHua QingGan" OR "Jin Hua Qing Gan" OR LianHuaQingWen OR "LianHua QingWen" OR "Lian Hua Qing Wen" OR XueBiJing OR "Xue Bi Jing" OR QingFeiPaiDu OR "QingFei PaiDu" OR "Qing Fei Pai Du" OR HuaShiBaiDu OR "HuaShi BaiDu" OR "Hua Shi Bai Du" OR XuanFeiBaiDu OR "XuanFei BaiDu" OR "Xuan Fei Bai Du"):ti,ab,kw |  |
|  | 6 | "alternative Medicine"/de OR "traditional medicine"/exp OR "plant medicinal product"/exp OR "phytotherapy"/exp OR "plant"/exp |  |
|  | 9 | (#1 OR #2 OR #3 OR #4) AND (#5 OR #6) AND [embase]/lim | **All: 7,347** |
|  | 10 | #9 AND ("randomized controlled trial"/de or "controlled clinical trial"/de or "randomization"/de or "intermethod comparison"/de or "double blind procedure"/de or "human experiment"/de **OR (**random* or placebo or assigned or allocated or volunteer or volunteers **or (**open NEXT/1 label**) or (**(double or single or doubly or singly) NEXT/1 (blind or blinded or blindly)**)** or "parallel group?" or crossover or "cross over" or **(**(assign* or match or matched or allocation) NEAR/5 (alternate or group? or intervention? or patient? or subject? or participant?)**) OR (**controlled NEAR/7 (study or design or trial)**)):ti,ab** **OR** **(**compare or compared or comparison or trial**):ti OR (**(evaluated or evaluate or evaluating or assessed or assess) and (compare or compared or comparing or comparison)**):ab) NOT** **((**(random* NEXT/1 sampl* NEAR/7 ("cross section*" or questionnaire? or survey* or database?)):ti,ab not ("comparative study"/de or "controlled study"/de or "randomi?ed controlled":ti,ab or "randomly assigned":ti,ab)**)** **OR (**"Cross-sectional study"/de not ("randomized controlled trial"/de or "controlled clinical study"/de or "controlled study"/de or randomi?ed controlled:ti,ab or "control group?":ti,ab)**) OR (**(((case NEXT/1 control*) and random*) not randomi?ed controlled):ti,ab**) OR** ("Systematic review" not (trial or study)):ti OR (nonrandom* not random*):ti,ab **OR** "Random field*":ti,ab **OR** ("random cluster" NEAR/3 sampl*):ti,ab **OR (**(review:ab and review/it) not trial:ti**)** **OR** **(**"we searched":ab and (review:ti or review/it)**) OR** "update review":ab **OR** (databases NEAR/4 searched):ab **OR (**(rat or rats or mouse or mice or swine or porcine or murine or sheep or lambs or pigs or piglets or rabbit or rabbits or cat or cats or dog or dogs or cattle or bovine or monkey or monkeys or trout or marmoset?):ti and "animal experiment"/de**) OR (**"animal experiment"/de not ("human experiment"/de or "human"/de)**))**  Filter Source: Box 3.e., [Technical Supplement to Chapter 4: Searching for and Selecting Studies.](https://training.cochrane.org/handbook/version-6/chapter-4-tech-suppl) Cochrane Handbook for Systematic Reviews of Interventions Version 6. (Syntax Translated from Ovid Embase to Elsevier Embase.com.) | **RCT: 813** |
| **MEDLINE (Ovid)** | 1 | (COVID OR "HCoV-19" OR "new coronavirus*" OR "novel coronavirus*" OR "SARS coronavirus* 2" OR "SARS-CoV-2" OR "SARS-CoV2" OR "SARS-CoV-II" OR "SARS 2" OR SARS2 OR nCoV OR nCOVID OR "severe acute respiratory syndrome coronavirus 2" OR "severe acute respiratory syndrome 2" OR "wuhan seafood market pneumonia virus*" OR ((wuhan OR chinese OR china) ADJ3 coronavirus*)).ti,ab,kf |  |
|  | 2 | exp "COVID-19"/ OR exp "sars-cov-2"/ |  |
|  | 3 | (coronavirus* OR COV OR sars OR "severe acute respiratory syndrome").ti,ab,kf |  |
|  | 4 | limit 3 to dt="20191101-20211231" |  |
|  | 5 | ("Coronaviridae Infections"/ OR "Coronavirus Infections"/ OR "Severe Acute Respiratory Syndrome"/ OR "Coronaviridae"/ OR "Coronavirus"/) |  |
|  | 6 | limit 5 to dt="20191101-20211231" |  |
|  | 7 | ((traditional ADJ4 medic*) OR TCM OR CAM OR herb* OR phytotherap* OR phytomedicine OR "chung i" OR "zhong yi" **OR** ((chinese OR orienta* OR kampo OR botanic* OR phyto* OR plant*) ADJ6 (drug* OR medic* OR formula* OR remed* OR preparation* OR capsule* OR pellet* OR solution* OR pill* OR powder* OR granule* OR granula* OR extract* OR product* OR agent* OR substance* OR material* OR traditional OR phyto* OR plant* OR therap* OR treatment* OR intervention* OR supplement*)**) OR (**(complementary OR alternative) ADJ6 (drug* OR medic* OR formula* OR remed* OR preparation* OR therap* OR treamment* OR intervention*)**) OR** fang OR tang OR wan OR san OR dan OR decoction* OR NRICM101 OR ChingGuan OR "Ching Guan" OR JinHuaQingGan OR "JinHua QingGan" OR "Jin Hua Qing Gan" OR LianHuaQingWen OR "LianHua QingWen" OR "Lian Hua Qing Wen" OR XueBiJing OR "Xue Bi Jing" OR QingFeiPaiDu OR "QingFei PaiDu" OR "Qing Fei Pai Du" OR HuaShiBaiDu OR "HuaShi BaiDu" OR "Hua Shi Bai Du" OR XuanFeiBaiDu OR "XuanFei BaiDu" OR "Xuan Fei Bai Du").ti,ab,kf |  |
|  | 8 | "Complementary Therapies"/ OR exp "Medicine, Traditional"/ OR exp "Phytotherapy"/ OR exp "Plant Preparations"/ OR exp "Herbal Medicine"/ OR exp "Plants"/ |  |
|  | 9 | (1 OR 2 OR 4 OR 6) AND (7 OR 8) | **All: 4,404** |
|  | 10 | 9 AND (("randomized controlled trial" or "controlled clinical trial").pt. or randomized.ab. or randomised.ab. or placebo.ab. or drug therapy.fs. or randomly.ab. or trial.ab. or groups.ab.) not (exp animals/ not humans.sh.)  Filter Source: Box 3.c., [Technical Supplement to Chapter 4: Searching for and Selecting Studies.](https://training.cochrane.org/handbook/version-6/chapter-4-tech-suppl) Cochrane Handbook for Systematic Reviews of Interventions Version 6. (Add: randomised.ab) | **RCT: 1,259** |
| **Cochrane Library** | 1 | (COVID OR "HCoV-19" OR "new coronavirus*" OR "novel coronavirus*" OR "SARS coronavirus* 2" OR "SARS-CoV-2" OR "SARS-CoV2" OR "SARS-CoV-II" OR "SARS 2" OR SARS2 OR nCoV OR nCOVID OR "severe acute respiratory syndrome coronavirus 2" OR "severe acute respiratory syndrome 2" OR "wuhan seafood market pneumonia virus*" OR ((wuhan OR chinese OR china) NEAR/2 coronavirus*)):ti,ab,kw  *Limits: with Publication Year from 2019 to present , in Trials* |  |
|  | 2 | [mh "COVID-19"] OR [mh "sars-cov-2"] |  |
|  | 3 | (coronavirus* OR COV OR sars OR "severe acute respiratory syndrome"):ti,ab,kw  *Limits: with Publication Year from 2019 to present , in Trials* |  |
|  | 4 | [mh ^"Coronaviridae Infections"] OR [mh ^"Coronavirus Infections"] OR [mh ^"Severe Acute Respiratory Syndrome"] OR [mh ^"Coronaviridae"] OR [mh ^"Coronavirus"/)  *Limits: with Publication Year from 2019 to present , in Trials* |  |
|  | 5 | ((traditional NEAR/3 medic*) OR TCM OR CAM OR herb* OR phytotherap* OR phytomedicine OR "chung i" OR "zhong yi" **OR** ((chinese OR orienta* OR kampo OR botanic* OR phyto* OR plant*) NEAR/6 (drug* OR medic* OR formula* OR remed* OR preparation* OR capsule* OR pellet* OR solution* OR pill* OR powder* OR granule* OR granula* OR extract* OR product* OR agent* OR substance* OR material* OR traditional OR phyto* OR plant* OR therap* OR treatment* OR intervention* OR supplement*)**) OR (**(complementary OR alternative) NEAR/6 (drug* OR medic* OR formula* OR remed* OR preparation* OR therap* OR treamment* OR intervention*)**) OR** fang OR tang OR wan OR san OR dan OR decoction* OR NRICM101 OR ChingGuan OR "Ching Guan" OR JinHuaQingGan OR "JinHua QingGan" OR "Jin Hua Qing Gan" OR LianHuaQingWen OR "LianHua QingWen" OR "Lian Hua Qing Wen" OR XueBiJing OR "Xue Bi Jing" OR QingFeiPaiDu OR "QingFei PaiDu" OR "Qing Fei Pai Du" OR HuaShiBaiDu OR "HuaShi BaiDu" OR "Hua Shi Bai Du" OR XuanFeiBaiDu OR "XuanFei BaiDu" OR "Xuan Fei Bai Du"):ti,ab,kw |  |
|  | 6 | [mh ^"Complementary Therapies"] OR [mh "Medicine, Traditional"] OR [mh "Phytotherapy"] OR [mh "Plant Preparations"] OR [mh "Herbal Medicine"] OR [mh "Plants"] |  |
|  | 7 | (#1 OR #2 OR #3 OR #4) AND (#5 OR #6) | **All: 488** |
|  | 8 | #7 Limits in Cochrane Reviews, Protocols, Trials | **SR+RCT: 480** |
| **Scopus** | 1 | **title-abs** (COVID OR "HCoV-19" OR "new coronavirus*" OR "novel coronavirus*" OR "SARS coronavirus* 2" OR "SARS-CoV-2" OR "SARS-CoV2" OR "SARS-CoV-II" OR "SARS 2" OR SARS2 OR nCoV OR nCOVID OR "severe acute respiratory syndrome coronavirus 2" OR "severe acute respiratory syndrome 2" OR "wuhan seafood market pneumonia virus*" OR ((wuhan OR chinese OR china) W/2 coronavirus*)) **OR authkey** (COVID OR "HCoV-19" OR "new coronavirus*" OR "novel coronavirus*" OR "SARS coronavirus* 2" OR "SARS-CoV-2" OR "SARS-CoV2" OR "SARS-CoV-II" OR "SARS 2" OR SARS2 OR nCoV OR nCOVID OR "severe acute respiratory syndrome coronavirus 2" OR "severe acute respiratory syndrome 2" OR "wuhan seafood market pneumonia virus*" OR ((wuhan OR chinese OR china) W/2 coronavirus*)) |  |
|  | 2 | **title-abs (**coronavirus* OR COV OR sars OR "severe acute respiratory syndrome"**) OR authkey (**coronavirus* OR COV OR sars OR "severe acute respiratory syndrome"**)**  *Limits:* |  |
|  | 3 | **title-abs (**coronavirus* OR COV OR sars OR "severe acute respiratory syndrome"**) OR authkey (**coronavirus* OR COV OR sars OR "severe acute respiratory syndrome"**)**  **AND**  ( LIMIT-TO ( PUBYEAR , 2023 ) OR LIMIT-TO ( PUBYEAR , 2022 ) OR LIMIT-TO ( PUBYEAR , 2021 ) OR LIMIT-TO ( PUBYEAR , 2020 ) OR LIMIT-TO ( PUBYEAR , 2019 ) ) |  |
|  | 4 | **title-abs** ((traditional W/3 medic*) OR TCM OR CAM OR herb* OR phytotherap* OR phytomedicine OR "chung i" OR "zhong yi" **OR** ((chinese OR orienta* OR kampo OR botanic* OR phyto* OR plant*) W/6 (drug* OR medic* OR formula* OR remed* OR preparation* OR capsule* OR pellet* OR solution* OR pill* OR powder* OR granule* OR granula* OR extract* OR product* OR agent* OR substance* OR material* OR traditional OR phyto* OR plant* OR therap* OR treatment* OR intervention* OR supplement*)**) OR (**(complementary OR alternative) W/6 (drug* OR medic* OR formula* OR remed* OR preparation* OR therap* OR treamment* OR intervention*)**) OR** fang OR tang OR wan OR san OR dan OR decoction* OR NRICM101 OR ChingGuan OR "Ching Guan" OR JinHuaQingGan OR "JinHua QingGan" OR "Jin Hua Qing Gan" OR LianHuaQingWen OR "LianHua QingWen" OR "Lian Hua Qing Wen" OR XueBiJing OR "Xue Bi Jing" OR QingFeiPaiDu OR "QingFei PaiDu" OR "Qing Fei Pai Du" OR HuaShiBaiDu OR "HuaShi BaiDu" OR "Hua Shi Bai Du" OR XuanFeiBaiDu OR "XuanFei BaiDu" OR "Xuan Fei Bai Du") **OR authkey** ((traditional W/3 medic*) OR TCM OR CAM OR herb* OR phytotherap* OR phytomedicine OR "chung i" OR "zhong yi" **OR** ((chinese OR orienta* OR kampo OR botanic* OR phyto* OR plant*) W/6 (drug* OR medic* OR formula* OR remed* OR preparation* OR capsule* OR pellet* OR solution* OR pill* OR powder* OR granule* OR granula* OR extract* OR product* OR agent* OR substance* OR material* OR traditional OR phyto* OR plant* OR therap* OR treatment* OR intervention* OR supplement*)**) OR (**(complementary OR alternative) W/6 (drug* OR medic* OR formula* OR remed* OR preparation* OR therap* OR treamment* OR intervention*)**) OR** fang OR tang OR wan OR san OR dan OR decoction* OR NRICM101 OR ChingGuan OR "Ching Guan" OR JinHuaQingGan OR "JinHua QingGan" OR "Jin Hua Qing Gan" OR LianHuaQingWen OR "LianHua QingWen" OR "Lian Hua Qing Wen" OR XueBiJing OR "Xue Bi Jing" OR QingFeiPaiDu OR "QingFei PaiDu" OR "Qing Fei Pai Du" OR HuaShiBaiDu OR "HuaShi BaiDu" OR "Hua Shi Bai Du" OR XuanFeiBaiDu OR "XuanFei BaiDu" OR "Xuan Fei Bai Du") |  |
|  | 5 | INDEXTERMS ( "clinical trials" OR "clinical trials as a topic" OR "randomized controlled trial" OR "Randomized Controlled Trials as Topic" OR "controlled clinical trial" OR "Controlled Clinical Trials" OR "random allocation" OR "Double-Blind Method" OR "Single-Blind Method" OR "Cross-Over Studies" OR "Placebos" OR "multicenter study" OR "double blind procedure" OR "single blind procedure" OR "crossover procedure" OR "clinical trial" OR "controlled study" OR "randomization" OR "placebo") OR TITLE-ABS-KEY ("clinical trials" OR "clinical trials as a topic" OR "randomized controlled trial" OR "Randomized Controlled Trials as Topic" OR "controlled clinical trial" OR "Controlled Clinical Trials as Topic" OR "random allocation" OR "randomly allocated" OR "allocated randomly" OR "Double-Blind Method" OR "Single-Blind Method" OR "Cross-Over Studies" OR "Placebos" OR "cross-over trial" OR "single blind" OR "double blind" OR "factorial design" OR "factorial trial") OR TITLE (clinical trial OR trial OR rct* OR random* OR blind*)  Filter Source: [This filter is built by NUS Medical Library](https://libguides.nus.edu.sg/c.php?g=145717&p=2470589) using MESH and EMTREE terms as Index terms and Keywords. Useful to search in Scopus for RCTs. |  |
|  | 5 | (#1 OR #3) AND #4 AND #5 | **RCT 1,306** |
| **CNKI**  [**https://tra.oversea.cnki.net/**](https://tra.oversea.cnki.net/) | 1 | SU = (新冠 + 新型冠狀 + 武漢肺炎 + COVID + "HCoV-19" + "new coronavirus" + "new coronaviruses" + "novel coronavirus" + "novel coronaviruses" + "SARS coronavirus 2" + "SARS coronaviruses 2" + "SARS-CoV-2" + "SARS-CoV2" + "SARS-CoV-II" + "SARS 2" + SARS2 + nCoV + nCOVID + "severe acute respiratory syndrome coronavirus 2" + "severe acute respiratory syndrome 2" + "wuhan seafood market pneumonia virus" + "wuhan seafood market pneumonia viruses") |  |
|  | 2 | Searched in result #1:  SU = (中醫 + 中西醫 + 中藥 + 中西藥 + 草藥 + 漢方 + 中成藥 + 方 + 湯 + 丸 + 散 + 粉 + 丹 + 清冠 + 金花輕感 + 連花清瘟 + 蓮花清瘟 + 血必淨 + 清肺排毒 + 化濕敗毒 + 化溼敗毒 + 宣肺敗毒 + TCM + CAM + herb + herbal + phytotherapy + phytotherapies + phytomedicine + phyto + "chung i" + "zhong yi" + botanic + botanical + plant + plants + complementary + fang + tang + wan + san + dan + decoction + decoctions + NRICM101 + ChingGuan + "Ching Guan" + JinHuaQingGan + "JinHua QingGan" + "Jin Hua Qing Gan" + LianHuaQingWen + "LianHua QingWen" + "Lian Hua Qing Wen" + XueBiJing + "Xue Bi Jing" + QingFeiPaiDu + "QingFei PaiDu" + "Qing Fei Pai Du" + HuaShiBaiDu + "HuaShi BaiDu" + "Hua Shi Bai Du" + XuanFeiBaiDu + "XuanFei BaiDu" + "Xuan Fei Bai Du") |  |
|  | 3 | Searched in result #2:  SU = (隨機 + 對照 + 控制組 + 安慰劑 + 試驗 + 分組 + random + randomly + randomized + randomized + placebo + trial + groups) | **RCT 147** |
| **Wanfang Data**  [**https://c.wanfangdata.com.cn/**](https://c.wanfangdata.com.cn/) |  | 主題: (("新冠" OR "新型冠狀" OR COVID OR "HCoV-19" OR "new coronavirus" OR "novel coronavirus" OR "SARS coronavirus 2" OR "SARS-CoV-2" OR "SARS-CoV2" OR "SARS-CoV-II" OR "SARS 2" OR SARS2 OR nCoV OR nCOVID) AND ("中醫" OR "中西醫" OR "中藥" OR "中西藥" OR "草藥" OR "漢方" OR "中成藥" OR "湯" OR "丸" OR "散" OR "粉" OR "丹" OR "清冠" OR "金花輕感" OR "連花清瘟" OR "蓮花清瘟" OR "血必淨" OR "清肺排毒" OR "化濕敗毒" OR "宣肺敗毒" OR TCM OR CAM OR herb* OR phyto* OR "chung i" OR "zhong yi" OR botanic* OR plant* OR complementary OR fang OR tang OR wan OR san OR dan OR decoction* OR NRICM101 OR ChingGuan OR "Ching Guan" OR JinHuaQingGan OR "JinHua QingGan" OR "Jin Hua Qing Gan" OR LianHuaQingWen OR "LianHua QingWen" OR "Lian Hua Qing Wen" OR XueBiJing OR "Xue Bi Jing" OR QingFeiPaiDu OR "QingFei PaiDu" OR "Qing Fei Pai Du" OR HuaShiBaiDu OR "HuaShi BaiDu" OR "Hua Shi Bai Du" OR XuanFeiBaiDu OR "XuanFei BaiDu" OR "Xuan Fei Bai Du") AND ("隨機" OR "對照" OR "控制組" OR "安慰" OR random* OR placebo*)) | **RCT 307** |
