# Supplementary material for: Therapeutic effects of herbal-medicine combined therapy for COVID-19: A systematic review and meta-analysis of randomized controlled trials
Source: Front Pharmacol. 2022 Sep 1;13:950012. doi: 10.3389/fphar.2022.950012 (PMC9475194; doi:10.3389/fphar.2022.950012)
Supplement: Supplementary file 3 [file Table2.docx]

| **Prescription/Main ingredient** | **composition** | **Preparation or details** |
| --- | --- | --- |
| Jinhua Qinggan Granules (China)  XueDong An 2021 | Lonicerae Japonicae Caulis(Caprifoliaceae, Lonicera japonica Thunb); Gypsum(Calcium sulfate dehydrate, Gypsum fibrosum); Ephedrae Herba (Ephedraceae, [Ephedra equisetina Bunge](https://mpns.science.kew.org/mpns-portal/plantDetail?plantId=332918&query=Ephedrae+Herba&filter=&fuzzy=false&nameType=all&dbs=wcs)); [armeniacae semen amarum](https://mpns.science.kew.org/mpns-portal/drugDetail?drugName=armeniacae+semen+amarum&query=Semen+Armeniacae+Amarum&filter=&fuzzy=false&nameType=all) (Rosaceae, *Prunus* armeniaca L.); Scutellaria baicalensis (Lamiaceae, [scutellariae baicalensis radix](https://mpns.science.kew.org/mpns-portal/drugDetail?drugName=scutellariae+baicalensis+radix&query=Scutellaria+baicalensis+&filter=&fuzzy=false&nameType=all)); Forsythia suspense (Oleaceae, Forsythia suspensa (Thunb.) Vahl); Fritillaria thunbergia (Liliaceae, Fritillaria thunbergii Miq.); Anemarrhena asphodeloides (Anemarrhena, [Anemarrhena asphodeloides Bunge](https://mpns.science.kew.org/mpns-portal/plantDetail?plantId=299155&query=Asphodeloides+Bunge&filter=&fuzzy=false&nameType=all&dbs=wcs)); Arctium lappa (Asteraceae, Arctium lappa L.); Artemisia annua(Asteraceae, [Artemisia annua L.](https://mpns.science.kew.org/mpns-portal/plantDetail?plantId=901442&query=Artemisia+annua&filter=&fuzzy=false&nameType=all&dbs=wcsCmp)); Mentha piperita (Lamiaceae, [Mentha × piperita L.](https://mpns.science.kew.org/mpns-portal/plantDetail?plantId=125188&query=Mentha+piperita&filter=&fuzzy=false&nameType=all&dbs=wcs)); Radix Glycyrrhizae(Fabaceae, [Glycyrrhiza uralensis Fisch. ex DC.](https://mpns.science.kew.org/mpns-portal/plantDetail?plantId=827776&query=Radix+Glycyrrhizae&filter=&fuzzy=false&nameType=all&dbs=wcsCmp)). The amount of each drug in a polyherbal preparation is unavailable in original text | Jinhua Qinggan granules (SFDA approval No.: Z20160001,  Juxiechang (Beijing) Pharmaceuticals Co., Ltd., Specification:  5 g/bag, Batch No.: 20200113  The extraction procedure has not been addressed in manuscript. |
| Maxingshigan-Weijing Decoction (China)  Congcong Zeng 2021 | Maxingshigan-Weijing decoction (MWD), which consists of 10 g of Ephedrae Herba (Ephedraceae, [Ephedra equisetina Bunge](https://mpns.science.kew.org/mpns-portal/plantDetail?plantId=332918&query=Ephedrae+Herba&filter=&fuzzy=false&nameType=all&dbs=wcs));10 g of [armeniacae semen amarum](https://mpns.science.kew.org/mpns-portal/drugDetail?drugName=armeniacae+semen+amarum&query=Semen+Armeniacae+Amarum&filter=&fuzzy=false&nameType=all) (Rosaceae, *Prunus* armeniaca L.); 45 g of Gypsum(Calcium sulfate dehydrate, Gypsum fibrosum), 30 g of Phragmitis Rhizoma (Poaceae, Phragmites australis (Cav.) Trin. ex Steud.), 20 g of peach kernel(Rosaceae, [Prunus persica (L.) Batsch](https://mpns.science.kew.org/mpns-portal/plantDetail?plantId=901279&query=peach+kernel&filter=&fuzzy=false&nameType=all&dbs=wcsCmp)); 20 g of , Benincasa hispida (Cucurbitaceae, [Benincasa hispida (Thunb.) Cogn.](https://mpns.science.kew.org/mpns-portal/plantDetail?plantId=673135&query=Benincasa+hispida+++&filter=&fuzzy=false&nameType=all&dbs=wcsCmp)); 30 g of Trichosanthes Kirilowii (Cucurbitaceae, Trichosanthes Kirilowii Maxim ); 12 g of Pericarpium Citri Reticulatae (Rutaceae, Citrus × aurantium L. ); 12 g of Pinelliae Rhizoma (Araceae, Pinellia ternata (Thunb.) Makino); 12 g of caulis bambusae in taeniis(Gramineae, [Bambusa tuldoides Munro](https://mpns.science.kew.org/mpns-portal/plantDetail?plantId=399090&query=caulis+bambusae+in+taeniis+&filter=&fuzzy=false&nameType=all&dbs=wcs)), 30 g of Lepidii seu Descurainiae Semen (Cruciferae, [Descurainia sophia (L.) Webb ex Prantl](https://mpns.science.kew.org/mpns-portal/plantDetail?plantId=761851&query=semen+lepidii+&filter=&fuzzy=false&nameType=all&dbs=wcsCmp)); 10 g of curcuma zedoary(Zingiberaceae, [Curcuma zedoaria](https://www.sciencedirect.com/topics/agricultural-and-biological-sciences/curcuma-zedoaria) Rosc. syn. C. zerumbet Roxb.) and 5 g of Glycyrrhizae Radix et Rhizoma (Fabaceae, [Glycyrrhiza glabra L.](https://mpns.science.kew.org/mpns-portal/plantDetail?plantId=827728&query=Liquorice+&filter=&fuzzy=false&nameType=all&dbs=wcsCmp)) | 200 mL of MWD orally 2 times daily for 14 consecutive days. The extraction procedure has not been addressed in manuscript. |
| Shuanghuanglian oral liquids (China)  Li Ni 2021 | Lonicerae Japonicae Caulis(Caprifoliaceae, Lonicera japonica Thunb);  Scutellaria baicalensis (Lamiaceae, Scutellaria baicalensis Georgi) and Forsythia suspense (Oleaceae, Forsythia suspensa (Thunb.) Vahl); | SHL (20 mL for the low-dose group, 40 mL for the middle dose group, and 60 mL for the high-dosegroup, three times daily) in addition to standard therapy or standard therapy alone for 14 days. (A Chinese patent medicine) |
| Xiyanping injection (XYP)(China)  Xin-Yi Zhang 2021 | Andrographis paniculate (Acanthaceae, Andrographis paniculata (Burm. F. ) Nees) The amount of each drug in a polyherbal preparation is unavailable | XYP injection(Jiangxi Qingfeng Pharmaceutical Co. Ltd) prepared from herbaceous plant A. paniculate. The bioactive ingredient, andrographolide, was extracted from the aerial part of A. paniculate using ethanol, and was then sulfonated through sulfonation reaction to generate XYP preparation. The concentration of andrographolide sulfonation product in XYP injection is 25 mg/ml. |
| shenhuang granule (China)  Shuang Zhou 2021 | 50g of Panax ginsen(Araliaceae, Panax ginseng C. A. Mey root); 40g of Rhei Radix et Rhizoma(Polygonaceae, [Rheum palmatum L.](https://mpns.science.kew.org/mpns-portal/plantDetail?plantId=425567&query=Rhei+Radix+et+Rhizoma&filter=&fuzzy=false&nameType=all&dbs=wcsCmp)); 30g of Sargentodoxa cuneata (Sargentodoxaceae, [Sargentodoxa cuneata (Oliv.) Rehder & E.H.Wilson](https://mpns.science.kew.org/mpns-portal/plantDetail?plantId=517495&query=Sargentodoxa+cuneata+&filter=&fuzzy=false&nameType=all&dbs=wcs)); 30g of Taraxaci Herba (Asteraceae, Taraxacum mongolicum Hand.-Mazz); 50g of Aconiti Lateralis Radix Praeparata (Ranunculaceae, [Raphanus raphanistrum subsp. sativus (L.) Domin](https://mpns.science.kew.org/mpns-portal/plantDetail?plantId=870724&query=Fuzi&filter=&fuzzy=false&nameType=all&dbs=wcsCmp)); 6g of Hirudo (Whitmania pigra Whitman) | After a series of extraction and manufacturing processes, the final product is a concentrated granule which is 1:5 of the raw herbs  The herbal medicine was extracted and manufacturing processed, the final product is a concentrated granule which is 1:5 of the raw herbs. The granules were packaged into two sachets before serving and dissolved in warm water and taken orally. |
| Huashi Baidu granule(China)  Zhijian Luo 2021 | 6g of Ephedrae Herba (Ephedraceae, [Ephedra equisetina Bunge](https://mpns.science.kew.org/mpns-portal/plantDetail?plantId=332918&query=Ephedrae+Herba&filter=&fuzzy=false&nameType=all&dbs=wcs)) ;9g of [armeniacae semen amarum](https://mpns.science.kew.org/mpns-portal/drugDetail?drugName=armeniacae+semen+amarum&query=Semen+Armeniacae+Amarum&filter=&fuzzy=false&nameType=all) (Rosaceae, Prunus armeniaca L.); 15 g of Gypsum(Calcium sulfate dehydrate, Gypsum fibrosum); 3g of Radix Glycyrrhizae(Fabaceae, [Glycyrrhiza uralensis Fisch. ex DC.](https://mpns.science.kew.org/mpns-portal/plantDetail?plantId=827776&query=Radix+Glycyrrhizae&filter=&fuzzy=false&nameType=all&dbs=wcsCmp)); 10g of Pogostemonis Herba (Lamiaceae, [Pogostemon cablin (Blanco) Benth.](https://mpns.science.kew.org/mpns-portal/plantDetail?plantId=162094&query=Pogostemonis+Herba&filter=&fuzzy=false&nameType=all&dbs=wcs)); 10g of Magnoliae Officinalis Cortex (Magnoliaceae, [Magnolia officinalis Rehder & E.H.Wilson](https://mpns.science.kew.org/mpns-portal/plantDetail?plantId=117741&query=Magnoliae+Officinalis+Cortex&filter=&fuzzy=false&nameType=all&dbs=wcs)); 15g of Atractylodis Rhizoma ( Compositae, atractylodis lanceae rhizoma ); 10g of Tsaoko Fructus (Zingiberaceae, Amomum tsaoko Crevost et Lemarie); 9g of Pinelliae Rhizoma (aroideae, [Pinellia ternata (Thunb.) Makino](https://mpns.science.kew.org/mpns-portal/plantDetail?plantId=156583&query=pinelliae+rhizoma&filter=&fuzzy=false&nameType=all&dbs=wcs)); 15g of poria, tuckahoe (Polyporaceae, Poria); 5g of Rhei Radix et Rhizoma (Rhubarb, [radix et rhizoma rhei](https://mpns.science.kew.org/mpns-portal/drugDetail?drugName=radix+et+rhizoma+rhei&query=Rhei+Radix+et+Rhizoma+++&filter=&fuzzy=false&nameType=all)); 10g of Astragali radix (Leguminosae, [Astragalus mongholicus Bunge](https://mpns.science.kew.org/mpns-portal/plantDetail?plantId=661222&query=Astragali+radix&filter=&fuzzy=false&nameType=all&dbs=wcsCmp)) ; 10g of Descurainiae Semen Lepidii Semen (Cruciferae, [Descurainia sophia (L.) Webb ex Prantl](https://mpns.science.kew.org/mpns-portal/plantDetail?plantId=761851&query=Descurainiae+Semen+Lepidii+Semen&filter=&fuzzy=false&nameType=all&dbs=wcsCmp)); 10g of Paeoniae Radix Rubra (Ranunculaceae, [Paeonia lactiflora Pall.](https://mpns.science.kew.org/mpns-portal/plantDetail?plantId=519125&query=Paeoniae+Radix+Rubra&filter=&fuzzy=false&nameType=all&dbs=wcs)) A Chinese patented medicine. <https://www.frontiersin.org/articles/10.3389/fmed.2021.696976/full#supplementary-material> | All Huashibaidu granule used within the trial was from the same batch and was donated by Huayi Pharmaceutical Co. Ltd. (Beijing, China) with Good Manufacturing Practice (GMP) qualification.  For treatment group, 20g of granules packed equally in two opaque bags. One bag of granules was to be dissolved in 150~200ml water at a temperature not lower than 80℃ and taken about 20min after breakfast and dinner. A Chinese patent medicine |
| Xuebijing injection (China)  Zhijian Luo 2021 | Paeoniae Radix Rubra (Ranunculaceae, [Paeonia lactiflora Pall.](https://mpns.science.kew.org/mpns-portal/plantDetail?plantId=519125&query=Paeoniae+Radix+Rubra&filter=&fuzzy=false&nameType=all&dbs=wcs)); Angelica Sinensis (Apiaceae, [angelicae sinensis radix](https://mpns.science.kew.org/mpns-portal/drugDetail?drugName=angelicae+sinensis+radix&query=Angelica+Sinensis&filter=&fuzzy=false&nameType=all)); Flos Carthami (Asteraceaec, [Carthamus tinctorius L.](https://mpns.science.kew.org/mpns-portal/plantDetail?plantId=900984&query=Flos+Carthami+&filter=&fuzzy=false&nameType=all&dbs=wcsCmp)) and Salvia miltiorrhiza Bunge  (Lamiaceae, Salviae miltiorrhizae radix et rhizome). The main ingredients of XBJ injection include amino acid, phenolic acid, flavonoid glycoside, elysine and phthalic acid ester. XBJ exerts effects on about 10 kinds of sepsis / inflammationpathways, and its 21 main active components participate in the regulation of 550 targets. Then XBJ injection possesses biological activity of promoting blood circulation and removing blood stasis and detoxifcation, therefore it is used in the treatment of sepsis with approval  The amount of each drug in a polyherbal preparation is unavailable in original text | XBJ group received routine medication plus XBJ injection, and 50 ml XBJ injection was diluted with 100 ml normal saline to 150 ml, every 12 h for 60 min. The manufacturer of XBJ injection was the Tianjin Chase Sun Pharmaceutical Co.,  Ltd., Tianjin, China (lot number Z20040033). A patent Chinese medicine |
| Xuanfei Baidu decoction (China)  Wu-zhong Xiong 2020 | 8g of Ephedrae Herba (Ephedraceae, [Ephedra equisetina Bunge](https://mpns.science.kew.org/mpns-portal/plantDetail?plantId=332918&query=Ephedrae+Herba&filter=&fuzzy=false&nameType=all&dbs=wcs)); 15g of [armeniacae semen amarum](https://mpns.science.kew.org/mpns-portal/drugDetail?drugName=armeniacae+semen+amarum&query=Semen+Armeniacae+Amarum&filter=&fuzzy=false&nameType=all) (Rosaceae, Prunus armeniaca L.); 30g of Gypsum(Calcium sulfate dehydrate, Gypsum fibrosum); 10g of Atractylodis Rhizoma (Compositae, [atractylodis lanceae rhizoma](https://mpns.science.kew.org/mpns-portal/drugDetail?drugName=atractylodis+lanceae+rhizoma&query=Atractylodis+Rhizoma+++&filter=&fuzzy=false&nameType=all)); 30g of Semen Coicis (Gramineae, [coicis semen](https://mpns.science.kew.org/mpns-portal/drugDetail?drugName=coicis+semen&query=Semen+Coicis&filter=&fuzzy=false&nameType=all)); 15g of , Agastachis Herba (Lamiaceae [herba agastachis](https://mpns.science.kew.org/mpns-portal/drugDetail?drugName=herba+agastachis&query=Agastachis+Herba+&filter=&fuzzy=false&nameType=all) ); 20g of Polygoni Cuspidati Rhizoma et Radix (Polygonaceae, [Reynoutria japonica Houtt.](https://mpns.science.kew.org/mpns-portal/plantDetail?plantId=428745&query=Polygoni+Cuspidati+Rhizoma+et+Radix&filter=&fuzzy=false&nameType=all&dbs=wcsCmp)); 15g of Lepidii seu Descurainiae Semen (Cruciferae, Descurainia sophia (L.) Webb ex Prantl); 30g of Verbenae Herba (Verbenaceae, [Verbena officinalis L.](https://mpns.science.kew.org/mpns-portal/plantDetail?plantId=212527&query=Verbenae+Herba+++&filter=&fuzzy=false&nameType=all&dbs=wcs) ); 30g of Phragmitis Rhizoma (Gramineae, [Phragmites australis (Cav.) Trin. ex Steud.](https://mpns.science.kew.org/mpns-portal/plantDetail?plantId=433921&query=Phragmitis+Rhizoma++++&filter=&fuzzy=false&nameType=all&dbs=wcs)); 25g of Artemisia carvifolia(Asteraceae, [Artemisia caruifolia var. caruifolia](https://mpns.science.kew.org/mpns-portal/plantDetail?plantId=1265755&query=Artemisia+carvifolia&filter=&fuzzy=false&nameType=all&dbs=wcsCmp)); 20g of Pericarpium Citri Reticulatae (Rutaceae, Citrus × aurantium L.); 10g of Glycyrrhizae Radix et Rhizoma (Glycyrrhizae, [Glycyrrhiza uralensis Fisch. ex DC.](https://mpns.science.kew.org/mpns-portal/plantDetail?plantId=827776&query=Glycyrrhizae+Radix+et+Rhizoma&filter=&fuzzy=false&nameType=all&dbs=wcsCmp)) | For treatment group, XBD (1 pouch of 200 ml each time, 2times/day) was added on top of conventional medicine which were treated to the control group. The treatment time for both groups were 1 week. |
| Reduning injection (China)  Xiaolong Xu 2021 | Artemisia annua (Asteraceae, Artemisia annua L); Lonicerae Japonicae Caulis (Caprifoliaceae, Lonicera japonica Thunb); and Gardenia jasminoides Ellis (Rubiaceae, Gardenia jasminoides J.Ellis ). Reduning injection is a Class II new TCM. It obtained a new drug certificate and production approval from the State Food and Drug Administration on May 10, 2005 (National Pharmaceutical Standard #Z20050217). | For Reduning treatment, 20 mL of Reduning injection was diluted with 250 mL of saline, and then intravenously infused once a day for 2 weeks. |
| Lianhua qingwen capsule(China)  Ke Hu 2021 | Forsythia suspensa（Oleaceae, Forsythia suspensa (Thunb.) Vahl); Lonicerae Japonicae Caulis(Caprifoliaceae, Lonicera japonica Thunb); Ephedrae Herba (Ephedraceae, [Ephedra equisetina Bunge](https://mpns.science.kew.org/mpns-portal/plantDetail?plantId=332918&query=Ephedrae+Herba&filter=&fuzzy=false&nameType=all&dbs=wcs)); Isatis indigotica (Brassicaceae, [Isatis tinctoria subsp. tinctoria](https://mpns.science.kew.org/mpns-portal/plantDetail?plantId=1265471&query=Isatis+indigotica+++&filter=&fuzzy=false&nameType=all&dbs=wcsCmp)); herba agastachis (Lamiaceae, Pogostemon cablin (Blanco) Benth. ); Rheum palmatum (Polygonaceae, [Rheum palmatum L.](https://mpns.science.kew.org/mpns-portal/plantDetail?plantId=425567&query=Rheum+palmatum&filter=&fuzzy=false&nameType=all&dbs=wcsCmp)); Glycyrrhiza uralensis (Leguminosae, [Glycyrrhiza uralensis Fisch. ex DC.](https://mpns.science.kew.org/mpns-portal/plantDetail?plantId=827776&query=Glycyrrhiza+uralensis&filter=&fuzzy=false&nameType=all&dbs=wcsCmp)); Dryopteris crassirhizoma (Dryopteridaceae, [Dryopteris crassirhizoma Nakai](https://mpns.science.kew.org/mpns-portal/plantDetail?plantId=902168&query=Dryopteris+crassirhizoma&filter=&fuzzy=false&nameType=all&dbs=wcsCmp)); Rhodiola crenulate (Crassulaceae, Rhodiola crenulata (Hook. f. et Thoms.) H. Ohba); Houttuynia cordata (Saururaceae, [Houttuynia cordata Thunb.](https://mpns.science.kew.org/mpns-portal/plantDetail?plantId=385534&query=Houttuynia+cordata&filter=&fuzzy=false&nameType=all&dbs=wcs)); Prunus sibirica (Rosaceae, [Prunus davidiana (Carrière) Franch.](https://mpns.science.kew.org/mpns-portal/plantDetail?plantId=901803&query=Prunus+sibirica&filter=&fuzzy=false&nameType=all&dbs=wcsCmp)); Gypsum(Calcium sulfate dehydrate, Gypsum fibrosum); A patented Chinese medicine The amount of each drug in a polyherbal preparation is unavailable in original text | 4 capsules thrice daily for treatment group.  Chinese medicine Z20100040, Beijing Yiling Pharmaceutical Co., Ltd., specification: 6 g per bag. |
| Lianhua Qingwen granules(China)  Huoxiang Zhengqi dropping pills(China)  Mingzhong Xiao 2020 | **Lianhua Qingwen granules:**  Forsythia suspensa（Oleaceae, Forsythia suspensa (Thunb.) Vahl); Lonicerae Japonicae Caulis(Caprifoliaceae, Lonicera japonica Thunb); Ephedrae Herba (Ephedraceae, [Ephedra equisetina Bunge](https://mpns.science.kew.org/mpns-portal/plantDetail?plantId=332918&query=Ephedrae+Herba&filter=&fuzzy=false&nameType=all&dbs=wcs)); Isatis indigotica (Brassicaceae, [Isatis tinctoria subsp. tinctoria](https://mpns.science.kew.org/mpns-portal/plantDetail?plantId=1265471&query=Isatis+indigotica+++&filter=&fuzzy=false&nameType=all&dbs=wcsCmp)); herba agastachis (Lamiaceae, Pogostemon cablin (Blanco) Benth. ); Rheum palmatum (Polygonaceae, [Rheum palmatum L.](https://mpns.science.kew.org/mpns-portal/plantDetail?plantId=425567&query=Rheum+palmatum&filter=&fuzzy=false&nameType=all&dbs=wcsCmp)); Glycyrrhiza uralensis (Leguminosae, [Glycyrrhiza uralensis Fisch. ex DC.](https://mpns.science.kew.org/mpns-portal/plantDetail?plantId=827776&query=Glycyrrhiza+uralensis&filter=&fuzzy=false&nameType=all&dbs=wcsCmp)); Dryopteris crassirhizoma (Dryopteridaceae, [Dryopteris crassirhizoma Nakai](https://mpns.science.kew.org/mpns-portal/plantDetail?plantId=902168&query=Dryopteris+crassirhizoma&filter=&fuzzy=false&nameType=all&dbs=wcsCmp)); Rhodiola crenulate (Crassulaceae, Rhodiola crenulata (Hook. f. et Thoms.) H. Ohba); Houttuynia cordata (Saururaceae, [Houttuynia cordata Thunb.](https://mpns.science.kew.org/mpns-portal/plantDetail?plantId=385534&query=Houttuynia+cordata&filter=&fuzzy=false&nameType=all&dbs=wcs)); Prunus sibirica (Rosaceae, [Prunus davidiana (Carrière) Franch.](https://mpns.science.kew.org/mpns-portal/plantDetail?plantId=901803&query=Prunus+sibirica&filter=&fuzzy=false&nameType=all&dbs=wcsCmp)); Gypsum(Calcium sulfate dehydrate, Gypsum fibrosum); A patented Chinese medicine  **Huoxiang Zhengqi dropping pills:** Pogostemonis Herba(Lamiaceae, Pogostemon cablin(Blanco) Benth); Atractylodes lancea(Asteraceae, Atractylodes lancea (Thunb.) DC.); Magnolia officinalis Cortex (Magnoliaceae, Magnolia officinalis Cortex [Magnolia officinalis Rehder & E.H.Wilson](https://mpns.science.kew.org/mpns-portal/plantDetail?plantId=117741&query=Magnolia+officinalis+Cortex&filter=&fuzzy=true&nameType=all&dbs=wcs)); Angelicae dahurica Radix (Umbelliferae, atractylodis lanceae rhizoma ); Poria (Polyporaceae, Poria cocos (Schw.) Wolf); Areca catechu(Arecaceae, Areca catechu L.); Pinellia ternata(Araceae, Pinellia ternate (Thunb.)); Glycyrrhizae Radix et Rhizoma (Fabaceae, extractum glycyrrhizae); Perillae Folium (Lamiaceae, Perilla frutescens (L.) Britton), and Citri Reticulatae Pericarpium (Rutaceae, Aurantii amari epicarpium et mesocarpium) The amount of each drug in a polyherbal preparation is unavailable in original text | **Lianhua Qingwen granules** (Chinese medicine Z20100040, Beijing Yiling Pharmaceutical Co., Ltd., specification: 6 g per bag).  **Huo Xiang Zhengqi dropping pills** (Chinese medicine Z20000048, Tianjin Tasly Pharmaceutical Group Co., Ltd, specification: 2.6 g in each bag)  Participants took one bag of Huoxiang Zhengqi dropping pills twice a day and one bag of Lianhua Qingwen granules three times a day. Subjects in Lianhua only group were treated with Lianhua Qingwen granules and western medicine, taking one bag of Lianhua Qingwen three times a day.  The drug quality standards for both medicines were complied with the provisions of Part I of the 2015 Edition of the Chinese Pharmacopoeia |
| Keguan-1 (China)  WANG Jia-bo 2020 | 30g of Lonicerae Japonicae Caulis(Caprifoliaceae, Lonicera japonica Thunb); 30g of Forsythia suspense (Oleaceae, Forsythia suspensa (Thunb.) Vahl); 15g of Folium Mori ( Moraceae, [Morus alba L.](https://mpns.science.kew.org/mpns-portal/plantDetail?plantId=501381&query=Folium+Mori&filter=&fuzzy=false&nameType=all&dbs=wcsCmp)); 10g of Chrysanthemi Flos (Asteraceae, Chrysanthemum morifolium Ramat.); 30g of Coicis Semen(Gramineae, Coix lacryma-jobi L. var. mayuen Stapf); 15g of Fritillariae Thunbergii Bulbus (Liliaceae, Fritillaria thunbergii Miq.) and 9g of Armeniacae Semen Amarum (Armeniacae, Prunusarmeniaca L. var. ansu Maxim.). Quality control assessments were based on the analyses of the relative amounts of the standard compounds by high-performance liquid chromatography tandem mass spectrometry (HPLC-MS) | The powder versions of the drugs for  the 7 components of Keguan-1 were obtained from Beijing Tcmages Pharmaceutical Co. Ltd. (Beijing,  China) and mixed in the defined ratio.  For the treatment group, each patient was given Keguan-1 19.4 g twice daily. |
| Qingfei Paidu decoction (China)  Wang, Q 2020 | 6g of Glycyrrhizae Radix et Rhizoma (Leguminosae, Glycyrrhiza uralensis); 9g of Ephedrae Herba (Ephedraceae, [Ephedra equisetina Bunge](https://mpns.science.kew.org/mpns-portal/plantDetail?plantId=332918&query=Ephedrae+Herba&filter=&fuzzy=false&nameType=all&dbs=wcs)); 15~30g of Gypsum(Calcium sulfate dehydrate, Gypsum fibrosum); 9g of [armeniacae semen amarum](https://mpns.science.kew.org/mpns-portal/drugDetail?drugName=armeniacae+semen+amarum&query=Semen+Armeniacae+Amarum&filter=&fuzzy=false&nameType=all) (Rosaceae, Prunus armeniaca L.); Poria (Polyporaceae, Poria cocos (Schw.) Wolf); 9g of Ramulus Cinnamomi (Camphor, cassiae cortex interior ), 9g of Atractylodes macrocephala (Asteraceae, [atractylodis macrocephalae rhizoma](https://mpns.science.kew.org/mpns-portal/drugDetail?drugName=atractylodis+macrocephalae+rhizoma&query=Atractylodes+macrocephala+++&filter=&fuzzy=false&nameType=all)); 9g of European Water Plantain(Alismataceae, Alisma plantago-aquatica [Alisma plantago-aquatica L.](https://mpns.science.kew.org/mpns-portal/plantDetail?plantId=294841&query=Alisma+plantago-aquatica&filter=&fuzzy=false&nameType=all&dbs=wcs)); 16g of Bupleuri Radix (Umbelliferae, Bupleurum falcatum L.); 15g of Poria (Polyporaceae, Poria cocos (Schw.) Wolf); 6g of Scutellaria baicalensis(Lamiaceae, Scutellaria baicalensis Georgi); 9g of Roof Iris (Iridaceae, Iris tectorum Maxim); 9g of Pinellia ternate(Araceae, Pinellia ternate (Thunb.)); 9g of Asteris Radix et Rhizoma (Asteraceae, [Aster tataricus L.f.](https://mpns.science.kew.org/mpns-portal/plantDetail?plantId=900882&query=Asteris+Radix+et+Rhizoma&filter=&fuzzy=false&nameType=all&dbs=wcsCmp)); 9g of Zingiberis Rhizoma (Zingiberaceae, Zingiber officinale Roscoe ); 9g of Agastache rugose(Lamiaceae, [Agastache rugosa (Fisch. & C.A.Mey.) Kuntze](https://mpns.science.kew.org/mpns-portal/plantDetail?plantId=4494&query=Agastache+rugose&filter=&fuzzy=true&nameType=all&dbs=wcs)); 6g of Trifoliate orange(Rutaceae, Citrus trifoliate L.); 6g of Citri Reticulatae Pericarpium (Rutaceae, [Citrus × aurantium L.](https://mpns.science.kew.org/mpns-portal/plantDetail?plantId=723957&query=Mandarin+orange&filter=&fuzzy=false&nameType=all&dbs=wcsCmp)); 6g of Asarum sieboldii (Aristolochiaceae, [radix et rhizoma asari](https://mpns.science.kew.org/mpns-portal/drugDetail?drugName=radix+et+rhizoma+asari&query=Asarum+sieboldii&filter=&fuzzy=false&nameType=all)); 12g of Dioscoreae Rhizoma (Dioscoreaceae, Dioscorea oppositifolia L.); 9g of Tussilago farfara (Asteraceae, Tussilago farfara L.) | Decoction was dissolved in 100mL warm water and taken after breakfast and dinner, |
| Shengmaisan (China)  He, Q., 2021 | Radix Ginseng (Araliaceae, Panax ginseng C.A.Mey ); Radix Ophiopogonis (Liliaceae, Ophiopogon japonicus (Thunb.) Ker Gawl.); Fructus Schisandrae (Magnoliaceae, Schisandra chinensis (Turcz.) Baill. ) The amount of each drug in a polyherbal preparation is unavailable in original text | A Chinese patent Medicine  one decoction daily and 0.5 pack bid. 7 consecutive days. |
| Buzhong Yiqi Decoction(China)  He, Q., 2021 | 10g of Astragali Radix (Fabaceae, Astragalus membranaceus); 3g of Panax ginseng (Araliaceae, Ginseng Radix et Rhizoma ); 5g of of Glycyrrhizae Radix et Rhizoma (Leguminosae, Glycyrrhiza uralensis); 3g of Citri Reticulatae Pericarpium (Rutaceae, [Citrus × aurantium L.](https://mpns.science.kew.org/mpns-portal/plantDetail?plantId=723957&query=Mandarin+orange&filter=&fuzzy=false&nameType=all&dbs=wcsCmp));3g of Angelica Sinensis (Apiaceae, Angelica sinensis (Oliv.) Diels ); 3g of Rhizoma Cimicifugae (Ranunculaceae, Actaea racemosa L. ); 3g of Bupleuri Radix (Umbelliferae, Bupleuri Radix L.) | A Chinese patent Medicine  one decoction daily and 0.5 pack bid. 7 consecutive days. |
| Fuzheng Gubiao Fangggan Decoction (China)  Zhang Dang 2021 | 12g of Astragalus membranaceus (Fabaceae, Astragalus mongholicus Bunge ); 9g Atractylodis Macrocephalae Rhizoma (Compositae, Atractylodes macrocephala Koidz); 6g of saposhnikovia root (Umbelliferae, Saposhnikoviae Radix); 12g of Poria (Polyporaceae, Poria cocos (Schw.) Wolf)); 6g of Citri Reticulatae Pericarpium (Rutaceae, [Citrus × aurantium L.](https://mpns.science.kew.org/mpns-portal/plantDetail?plantId=723957&query=Mandarin+orange&filter=&fuzzy=false&nameType=all&dbs=wcsCmp)); 9g of Forsythia suspense (Oleaceae, Forsythia suspensa (Thunb.) Vahl); 10g of Lonicerae Japonicae Caulis(Caprifoliaceae, Lonicera japonica Thunb); 6g of Perillae Folium (Lamiaceae, Perilla frutescens (L.) Britton); 3g of Glycyrrhizae Radix et Rhizoma (Leguminosae, Glycyrrhiza uralensis); 15g of Coicis Semen(Gramineae, Coix lacryma-jobi L. var. mayuen Stapf); 10g of Agastache rugose(Lamiaceae, [Agastache rugosa (Fisch. & C.A.Mey.) Kuntze](https://mpns.science.kew.org/mpns-portal/plantDetail?plantId=4494&query=Agastache+rugose&filter=&fuzzy=true&nameType=all&dbs=wcs)); 9g of Platycodon grandifloras (Campanulaceae, [Platycodon grandiflorus (Jacq.) A.DC.](https://mpns.science.kew.org/mpns-portal/plantDetail?plantId=354641&query=Platycodon+grandifloras&filter=&fuzzy=true&nameType=all&dbs=wcs)) | one decoction daily, add 150~200Ml of warm water into decoction, divide it into two part and take after breakfast and dinner for 7 consecutive days. |
| Jiawei Yupingfeng powder (China)  Ping Xianghua 2021 | 30g of Astragalus membranaceus (Fabaceae, [Astragalus mongholicus Bunge](https://mpns.science.kew.org/mpns-portal/plantDetail?plantId=661222&query=Astragalus+membranaceus&filter=&fuzzy=false&nameType=all&dbs=wcsCmp)); 12g of Atractylodis Macrocephalae Rhizoma (Compositae, Atractylodes macrocephala Koidz); 10g of Saposhnikoviae Radix (Umbelliferae, Saposhnikovia divaricata (Turcz. ex Ledeb.) Schischk.); 10g of Smilacis Glabrae Rhizoma (Smilacaceae, rhizoma smilacis glabrae); 10g of Rhizoma Atractylodis (Compositae, Rhizoma Atractylodis,Atractylodes lancea (Thunb.) DC.); 10g of Agastache rugose(Lamiaceae, Agastache rugose); 10g of Perillae Folium (Lamiaceae, Perilla frutescens (L.) Britton); 8g of Rhizoma Pinelliae (Araceae, Pinellia ternata (Thunb.) Makino) ; 6g of Wurfbainia villosa (Zingiberaceae, Wurfbainia villosa (Lour.) Skornick. & A.D.Poulsen ); 6g of Zingiber officinale (Zingiberaceae, [Zingiber officinale Roscoe](https://mpns.science.kew.org/mpns-portal/plantDetail?plantId=273361&query=Zingiber+officinale&filter=&fuzzy=false&nameType=all&dbs=wcs)) | one decoction daily and divide decoction into two part. Participants take one part after breakfast and dinner |
| Modified Shengjiang Powder(China)  YE Ling, 2021 | 10g of Bombyx Batryticatus (Bombycidae, Bombyx Batryticatus); 4g of Periostracum Cicadae (Cicadidae, Periostracum Cicadae); 8g of Curcuma longa (Zingiberaceae, [Curcuma longa L.](https://mpns.science.kew.org/mpns-portal/plantDetail?plantId=235249&query=Curcuma+longa&filter=&fuzzy=false&nameType=all&dbs=wcs)); 4g of Rhei Radix et Rhizoma (Rhubarb, Rheum palmatum L. ); 30g of Astragalus membranaceus (Fabaceae, Astragalus mongholicus Bunge ); 15g of Atractylodis Macrocephalae Rhizoma (Compositae, [Atractylodes macrocephala Koidz.](https://mpns.science.kew.org/mpns-portal/plantDetail?plantId=872479&query=Atractylodis+Macrocephalae+Rhizoma+++&filter=&fuzzy=false&nameType=all&dbs=wcsCmp)); 9g of saposhnikovia root (Umbelliferae, Saposhnikoviae Radix); 15g of Polygonum tinctorium Lour (Polygonaceae, [Persicaria tinctoria (Aiton) Spach](https://mpns.science.kew.org/mpns-portal/plantDetail?plantId=904240&query=Polygonum+tinctorium+Lour&filter=&fuzzy=false&nameType=all&dbs=wcsCmp)); 15g of Houttuynia cordata (Saururaceae, Houttuynia cordata Thunb); 9g of Agastache rugose(Lamiaceae, [Agastache rugosa (Fisch. & C.A.Mey.) Kuntze](https://mpns.science.kew.org/mpns-portal/plantDetail?plantId=4494&query=Agastache+rugose&filter=&fuzzy=true&nameType=all&dbs=wcs)) | one decoction daily and divide decoction into two part. Participants take one part after breakfast and dinner. |
| Antivirus No. 1(China)  Ling Chao 2021 | 15g of Rhizoma Atractylodis (Compositae, [Atractylodes lancea (Thunb.) DC.](https://mpns.science.kew.org/mpns-portal/plantDetail?plantId=900927&query=Rhizoma+Atractylodis+++++&filter=&fuzzy=false&nameType=all&dbs=wcsCmp)); 10g of Citri Reticulatae Pericarpium (Rutaceae, [Citrus × aurantium L.](https://mpns.science.kew.org/mpns-portal/plantDetail?plantId=723957&query=Mandarin+orange&filter=&fuzzy=false&nameType=all&dbs=wcsCmp)); 10g of Houpoea officinalis (Magnoliaceae, Magnolia officinalis Rehder & E.H.Wilson); 10g of Agastache rugose(Lamiaceae, Agastache rugose); 15g of Amomum tsao-ko (Zingiberaceae, Amomum tsaoko Crevost et Lemarie); 5g of Ephedrae Herba (Ephedraceae, [Ephedra equisetina Bunge](https://mpns.science.kew.org/mpns-portal/plantDetail?plantId=332918&query=Ephedrae+Herba&filter=&fuzzy=false&nameType=all&dbs=wcs)); 10g of Zingiber officinale (Zingiberaceae, [Zingiber officinale Roscoe](https://mpns.science.kew.org/mpns-portal/plantDetail?plantId=273361&query=Zingiber+officinale&filter=&fuzzy=false&nameType=all&dbs=wcs)) ;10g of [armeniacae semen amarum](https://mpns.science.kew.org/mpns-portal/drugDetail?drugName=armeniacae+semen+amarum&query=Semen+Armeniacae+Amarum&filter=&fuzzy=false&nameType=all) (Rosaceae, Prunus armeniaca L.);10g of Amomum verum (Zingiberaceae, [Wurfbainia vera (Blackw.) Skornick. & A.D.Poulsen](https://mpns.science.kew.org/mpns-portal/plantDetail?plantId=532064&query=Amomum+verum+&filter=&fuzzy=false&nameType=all&dbs=wcs)); 15g of Coicis Semen(Gramineae, Coix lacryma-jobi L. var. mayuen Stapf); 20g of Astragalus(Fabaceae, Astragalus membranaceus); 15g of Abrus mollis (Fabaceae, Abrus melanospermus subsp. melanospermus ); 15g of Rhizoma Pinelliae (Araceae, Pinellia ternata (Thunb.) Makino); 20g of Radix Ginseng (Araliaceae, Panax ginseng C.A.Mey ). | one decoction daily, herbal medicine was decocted in 450mL of water and  taken 150mL three times a day |
| Jiawei Sang Ju drink(China)  Liu, A.2021 | 20g of Folium Mori (Moraceae, Folium Mori [Abrus melanospermus subsp. melanospermus](https://mpns.science.kew.org/mpns-portal/plantDetail?plantId=1240347&query=Abrus+mollis+++&filter=&fuzzy=false&nameType=all&dbs=wcsCmp) ); 6g of Chrysanthemum (Compositae, [Matricaria chamomilla L.](https://mpns.science.kew.org/mpns-portal/plantDetail?plantId=906535&query=Chrysanthemum&filter=&fuzzy=false&nameType=all&dbs=wcsCmp) ); 20g of [armeniacae semen amarum](https://mpns.science.kew.org/mpns-portal/drugDetail?drugName=armeniacae+semen+amarum&query=Semen+Armeniacae+Amarum&filter=&fuzzy=false&nameType=all)(Rosaceae, Prunus armeniaca L.);10g of Platycodon grandifloras (Campanulaceae, Platycodon grandifloras Platycodon grandiflorus (Jacq.) A.DC. ); 15g of Forsythia suspense (Oleaceae, Forsythia suspensa (Thunb.) Vahl); 15g of Lonicerae Japonicae Caulis(Caprifoliaceae, Lonicera japonica Thunb); 6g of Mentha haplocalyx Briquet (Lamiaceae, Mentha canadensis L.); 15g of Fritillaria przewalkii Maxim (Liliaceae, Fritillaria przewalskii Maxim. ex Batalin); 6g Anemarrhena asphodeloides (Asparagaceae, Anemarrhena asphodeloides [Anemarrhena asphodeloides Bunge](https://mpns.science.kew.org/mpns-portal/plantDetail?plantId=299155&query=Anemarrhena+asphodeloides+++&filter=&fuzzy=false&nameType=all&dbs=wcs) ); 6g [Glycyrrhiza uralensis Fisch. ex DC.](https://mpns.science.kew.org/mpns-portal/plantDetail?plantId=827776&query=Glycyrrhiza+uralensis&filter=&fuzzy=false&nameType=all&dbs=wcsCmp)(Leguminosae, Glycyrrhiza uralensis); 10g of Phragmitis Rhizoma. (Gramineae, Phragmitis Rhizoma Phragmites australis (Cav.) Trin. ex Steud. ); 10g of Coicis Semen(Gramineae, Coix lacryma-jobi L. var. mayuen Stapf); 10g of medicated leaven (Depressariidae, Massa Medicata Fermentata); 10g of Crataegus pinnatifida (Rosaceae, [Crataegus monogyna Jacq.](https://mpns.science.kew.org/mpns-portal/plantDetail?plantId=921984&query=Crataegus+pinnatifida&filter=&fuzzy=false&nameType=all&dbs=wcsCmp)); 10g of Hordeum vulgare (Poaceae, Hordeum vulgare L.) | one decoction daily, divide decoction into three part and wait 30min to take 170mL after meal |
| Fang Shengmai Powder and Shenling Baizhu Powder (China)  WANG Lin 2020 | 30g of Pseudostellaria heterophylla (Caryophyllaceae, [Pseudostellaria heterophylla (Miq.) Pax](https://mpns.science.kew.org/mpns-portal/plantDetail?plantId=409729&query=Pseudostellaria+heterophylla&filter=&fuzzy=false&nameType=all&dbs=wcsCmp) ); 15g of Ophiopogonis Radix(Liliaceae, [Ophiopogon japonicus (Thunb.) Ker Gawl.](https://mpns.science.kew.org/mpns-portal/plantDetail?plantId=279475&query=Ophiopogonis+Radix&filter=&fuzzy=false&nameType=all&dbs=wcs) ); 15g of Schisandra(Schisandraceae, Schisandra chinensis [Schisandra chinensis (Turcz.) Baill.](https://mpns.science.kew.org/mpns-portal/plantDetail?plantId=381262&query=Schisandra+chinensis&filter=&fuzzy=false&nameType=all&dbs=wcs)); 15g of Poria (Polyporaceae, Poria cocos (Schw.) Wolf)); 10g of of Citri Reticulatae Pericarpium (Rutaceae, [Citrus × aurantium L.](https://mpns.science.kew.org/mpns-portal/plantDetail?plantId=723957&query=Mandarin+orange&filter=&fuzzy=false&nameType=all&dbs=wcsCmp)); 20g of Dioscorea batatas (Dioscoreaceae, [Dioscorea polystachya Turcz.](https://mpns.science.kew.org/mpns-portal/plantDetail?plantId=240732&query=Dioscorea+batatas&filter=&fuzzy=false&nameType=all&dbs=wcs)); 10g of Platycodon grandifloras (Campanulaceae, [Platycodon grandiflorus (Jacq.) A.DC.](https://mpns.science.kew.org/mpns-portal/plantDetail?plantId=354641&query=Platycodon+grandifloras&filter=&fuzzy=true&nameType=all&dbs=wcs)); 10g of Houpoea officinalis (Magnoliaceae, [Magnolia officinalis Rehder & E.H.Wilson](https://mpns.science.kew.org/mpns-portal/plantDetail?plantId=117741&query=Houpoea+officinalis&filter=&fuzzy=false&nameType=all&dbs=wcs) ); 15g of Rehmannia glutinosa Liboschitz (Orobanchaceae, Rehmannia glutinosa Liboschitz [Rehmannia glutinosa (Gaertn.) DC.](https://mpns.science.kew.org/mpns-portal/plantDetail?plantId=527243&query=Rehmannia+glutinosa+Liboschitz+++&filter=&fuzzy=false&nameType=all&dbs=wcsCmp)); 10g of Scrophularia ningpoensis (Scrophulariaceae, Scrophularia ningpoensis Hemsl. ); 20g of Salvia Miltiorrhiza (Lamiaceae, [Salvia miltiorrhiza Bunge](https://mpns.science.kew.org/mpns-portal/plantDetail?plantId=183206&query=Salvia+Miltiorrhiza++&filter=&fuzzy=false&nameType=all&dbs=wcs)); 10g of Glycyrrhiza uralensis (Leguminosae, [Glycyrrhiza uralensis Fisch. ex DC.](https://mpns.science.kew.org/mpns-portal/plantDetail?plantId=827776&query=Glycyrrhiza+uralensis&filter=&fuzzy=false&nameType=all&dbs=wcsCmp)) | Herbal medicine was decocted in 200mL of water and divided into two parts. Participants taken one part after breakfast and dinner.  A Chinese patent Medicine |
| Gegen Qinlian pill (China)  Wang, L 2020 | Radix Puerariae (leguminosae, [Pueraria montana var. lobata (Willd.) Maesen & S.M.Almeida ex Sanjappa & Predeep](https://mpns.science.kew.org/mpns-portal/plantDetail?plantId=537348&query=Radix+Puerariae&filter=&fuzzy=false&nameType=all&dbs=wcsCmp)); Scutellaria baicalensis Georgi (Lamiaceae, [Scutellaria baicalensis Georgi](https://mpns.science.kew.org/mpns-portal/plantDetail?plantId=188938&query=Scutellaria+baicalensis+Georgi&filter=&fuzzy=false&nameType=all&dbs=wcs)); Coptis chinensis(Ranunculaceae, Coptis chinensis Franch. ); Glycyrrhiza uralensis (Leguminosae, [Glycyrrhiza uralensis Fisch. ex DC.](https://mpns.science.kew.org/mpns-portal/plantDetail?plantId=827776&query=Glycyrrhiza+uralensis&filter=&fuzzy=false&nameType=all&dbs=wcsCmp)) The amount of each drug in a polyherbal preparation is unavailable in original text | 3g/pouch, three times daily and one pouch each time  A Chinese patent Medicine |
| Modified Maxing Shigan Sanren Decoction  (China)  Mou, F 2020 | Ephedrae Herba (Ephedraceae, [Ephedra equisetina Bunge](https://mpns.science.kew.org/mpns-portal/plantDetail?plantId=332918&query=Ephedrae+Herba&filter=&fuzzy=false&nameType=all&dbs=wcs)); [armeniacae semen amarum](https://mpns.science.kew.org/mpns-portal/drugDetail?drugName=armeniacae+semen+amarum&query=Semen+Armeniacae+Amarum&filter=&fuzzy=false&nameType=all)  (Rosaceae, Prunus armeniaca L.); Gypsum(Calcium sulfate dehydrate, Gypsum fibrosum);Glycyrrhiza uralensis (Leguminosae, [Glycyrrhiza uralensis Fisch. ex DC.](https://mpns.science.kew.org/mpns-portal/plantDetail?plantId=827776&query=Glycyrrhiza+uralensis&filter=&fuzzy=false&nameType=all&dbs=wcsCmp)); Coicis Semen(Gramineae, Coix lacryma-jobi L. var. mayuen Stapf); Wurfbainia vera (Zingiberaceae, Wurfbainia vera (Blackw.) Skornick. & A.D.Poulsen ); Bupleuri Radix (Umbelliferae, [Bupleurum falcatum L.](https://mpns.science.kew.org/mpns-portal/plantDetail?plantId=686570&query=Bupleuri+Radix&filter=&fuzzy=false&nameType=all&dbs=wcsCmp)); Scutellaria baicalensis Georgi (Lamiaceae, [Scutellaria baicalensis Georgi](https://mpns.science.kew.org/mpns-portal/plantDetail?plantId=188938&query=Scutellaria+baicalensis+Georgi&filter=&fuzzy=false&nameType=all&dbs=wcs)); Artemisiae Annuae(Asteraceae, Herba Artemisiae Annuae); bombyx batryticatus (Bombycidae, bombyx batryticatus); Periostracum Cicadae (Cicadidae,Periostracum Cicadae) The amount of each drug in a polyherbal preparation is unavailable in original text | Decoction was divided into 3 part and taken one part three times daily |
| Viable Bifidobacterium Tablets Combined with Sanren Decoction (China)  Shuang Wang 2020 | **Sanren Decotion**: 9g of Rhizoma Pinelliae (Araceae, Pinellia ternata (Thunb.) Makino ); 24g of Lophatherum gracile (Poaceae, [Lophatherum gracile Brongn.](https://mpns.science.kew.org/mpns-portal/plantDetail?plantId=422947&query=Lophatherum+gracile&filter=&fuzzy=false&nameType=all&dbs=wcs)); 3g of Tetrapanax papyrifer (Araliaceae, [Tetrapanax papyrifer (Hook.) K.Koch](https://mpns.science.kew.org/mpns-portal/plantDetail?plantId=202449&query=Tetrapanax+papyrifer&filter=&fuzzy=false&nameType=all&dbs=wcs) ); 12g of [armeniacae semen amarum](https://mpns.science.kew.org/mpns-portal/drugDetail?drugName=armeniacae+semen+amarum&query=Semen+Armeniacae+Amarum&filter=&fuzzy=false&nameType=all)(Rosaceae, Prunus armeniaca L.); 45g of Coicis Semen(Gramineae, Coix lacryma-jobi L. var. mayuen Stapf); 6g of Bupleuri Radix (Umbelliferae, [Bupleurum falcatum L.](https://mpns.science.kew.org/mpns-portal/plantDetail?plantId=686570&query=Bupleuri+Radix&filter=&fuzzy=false&nameType=all&dbs=wcsCmp)); 24g of Scutellaria baicalensis(Lamiaceae, Scutellaria baicalensis Georgi); 24g of Pseudostellaria heterophylla (Caryophyllaceae, [Pseudostellaria heterophylla (Miq.) Pax](https://mpns.science.kew.org/mpns-portal/plantDetail?plantId=409729&query=Pseudostellaria+heterophylla&filter=&fuzzy=false&nameType=all&dbs=wcsCmp)); 24g of Curcumae Radix (Zingiberaceae, [Curcuma longa L.](https://mpns.science.kew.org/mpns-portal/plantDetail?plantId=235249&query=Curcumae+Radix&filter=&fuzzy=false&nameType=all&dbs=wcs)); 45g of Poria (Polyporaceae, Poriacocos (Schw.) Wolf)); 24g of Fructus Citri Sarcodactylis (Rutaceae, Citrus medica L.); 45g of gramineae, Phragmitis Rhizoma (Phragmites australis (Cav.) Trin. ex Steud. ) | One decoction daily for 15 consecutive days |
| Maxing Xuanfei Jiedu Decoction (China)  Qiu Min 2020 | 9g of Ephedrae Herba (Ephedraceae, [Ephedra equisetina Bunge](https://mpns.science.kew.org/mpns-portal/plantDetail?plantId=332918&query=Ephedrae+Herba&filter=&fuzzy=false&nameType=all&dbs=wcs)); 12g of [armeniacae semen amarum](https://mpns.science.kew.org/mpns-portal/drugDetail?drugName=armeniacae+semen+amarum&query=Semen+Armeniacae+Amarum&filter=&fuzzy=false&nameType=all)(Rosaceae, Prunus armeniaca L.); 15~30g of Gypsum(Calcium sulfate dehydrate, Gypsum fibrosum); 12g of Bulbus Fritillariae Thunbergii (Lilium, Fritillaria thunbergii Miq. ); 10g of Periostracum Cicadae (Cicadidae, Periostracum Cicadae); 15g of Bombyx Batryticatus (Bombycidae, Bombyx Batryticatus); 12g of [Curcuma longa L.](https://mpns.science.kew.org/mpns-portal/plantDetail?plantId=235249&query=Curcuma+longa&filter=&fuzzy=false&nameType=all&dbs=wcs)(Zingiberaceae, Curcuma longa L. ); 12g of Platycodon(Campanulaceae, Platycodon grandifloras); 12g of Zhi Shi(Rutaceae, Fructus Aurantii); 9g of Tsaoko Fructus (Zingiberaceae, Amomum tsaoko Crevost et Lemarie ); 12g of Amomum verum(Zingiberaceae, Wurfbainia vera) | Herbal medicine was decocted twice, and divided into three parts, 150mL/part. Participants take one part three times daily. |
| Compound Yin Chai granule + Qingqiao detoxification granule  (China)  Jin W et al 2020 | **Yin Chai granule**: Lonicerae Japonicae Caulis(Caprifoliaceae, Lonicera japonica Thunb); Bupleuri Radix (Umbelliferae, [Bupleurum falcatum L.](https://mpns.science.kew.org/mpns-portal/plantDetail?plantId=686570&query=Bupleuri+Radix&filter=&fuzzy=false&nameType=all&dbs=wcsCmp)); Arundo (Poaceae, [Imperata cylindrica (L.) P.Beauv.](https://mpns.science.kew.org/mpns-portal/plantDetail?plantId=420125&query=Arundo+&filter=&fuzzy=false&nameType=all&dbs=wcs) ) ; Folium Eriobotryae (Rosaceae, Rhaphiolepis bibas (Lour.) Galasso & Banfi ); Mentha haplocalyx Briquet(Lamiaceae, Mentha canadensis L. ); Agastache rugose(Lamiaceae, Agastache rugose); [Nepeta tenuifolia Benth.](https://mpns.science.kew.org/mpns-portal/plantDetail?plantId=135025&query=Herba+Schizonepetae+++&filter=&fuzzy=false&nameType=all&dbs=wcs)(labiatae, Herba Schizonepetae )  **Qingqiao detoxification granule:** Lonicerae Japonicae Caulis(Caprifoliaceae, Lonicera japonica Thunb); Forsythia suspense (Oleaceae, Forsythia suspensa (Thunb.) Vahl); Podalirius thomsonii(legume, Pueraria lobata var. thomsonii); Angelica Dahurica (Apiaceae, Angelica dahurica (Hoffm.) Benth. & Hook.f. ex Franch. & Sav.); Artemisiae Annuae(Asteraceae, Herba Artemisiae Annuae); Bupleuri Radix (Umbelliferae, [Bupleurum falcatum L.](https://mpns.science.kew.org/mpns-portal/plantDetail?plantId=686570&query=Bupleuri+Radix&filter=&fuzzy=false&nameType=all&dbs=wcsCmp)); Paris(Melanthiaceae, Paris quadrifolia); Radix Isatidis (Cruciferae, [Isatis tinctoria L.](https://mpns.science.kew.org/mpns-portal/plantDetail?plantId=867046&query=Radix+Isatidis&filter=&fuzzy=false&nameType=all&dbs=wcsCmp)); Roof Iris(Iridaceae, Iris tectorum Maxim); Pugongying(Asteraceae, Taraxacum mongolicum whole plant); Indigoplant Leaf(Polygonaceae, Polygonum tinctorium Lour.); Agastache rugose(Lamiaceae,Agastache rugose); Perilla frutescens (Lamiaceae,Perilla frutescens [Perilla frutescens (L.) Britton](https://mpns.science.kew.org/mpns-portal/plantDetail?plantId=150299&query=Perilla+frutescens&filter=&fuzzy=false&nameType=all&dbs=wcs)); The amount of each drug in a polyherbal preparation is unavailable in original text | Participants take 15g of granule each time, 4 times daily. |
| Toujie Quwen Granules  (China)  FU Xiaoxia 2020 | 30g of Forsythia suspense (Oleaceae, Forsythia suspensa (Thunb.) Vahl); 20g of Cremastrae seu Pleiones Pseudobulbus (Orchidaceae, Cremastrae Pseudobulbus Pleiones Pseudobulbus); 15g of Lonicerae Japonicae Caulis(Caprifoliaceae, Lonicera japonica Thunb); 10g of Baikal skullcap(Lamiaceae, Scutellaria baicalensis Georgi); 10g of Polygonum tinctorium Lour (Polygonaceae, [Persicaria tinctoria (Aiton) Spach](https://mpns.science.kew.org/mpns-portal/plantDetail?plantId=904240&query=Polygonum+tinctorium+Lour&filter=&fuzzy=false&nameType=all&dbs=wcsCmp)); 5g of Bupleuri Radix (Umbelliferae, [Bupleurum falcatum L.](https://mpns.science.kew.org/mpns-portal/plantDetail?plantId=686570&query=Bupleuri+Radix&filter=&fuzzy=false&nameType=all&dbs=wcsCmp)) ; 10g of Artemisiae Annuae(Asteraceae, Artemisia annua L. ); 10g of Periostracum Cicadae (Cicadidae, Periostracum Cicadae); 5g of Peucedani Radix (Umbelliferae, [Kitagawia praeruptora (Dunn) Pimenov](https://mpns.science.kew.org/mpns-portal/plantDetail?plantId=1015229&query=Peucedani+Radix&filter=&fuzzy=false&nameType=all&dbs=wcsCmp)); 10g of Fritillaria przewalkii (Liliaceae, [Fritillaria przewalskii Maxim. ex Batalin](https://mpns.science.kew.org/mpns-portal/plantDetail?plantId=306829&query=Fritillaria+przewalkii&filter=&fuzzy=true&nameType=all&dbs=wcs)); 30g of Fructus Mume (Rosaceae, Prunus mume (Siebold) Siebold & Zucc. ); 45g of Astragalus membranaceus (Fabaceae, Astragalus mongholicus Bunge); 30g of Poria (Polyporaceae, Poria cocos (Schw.) Wolf)); 15g of Pseudostellaria heterophylla (Caryophyllaceae, [Pseudostellaria heterophylla (Miq.) Pax](https://mpns.science.kew.org/mpns-portal/plantDetail?plantId=409729&query=Pseudostellaria+heterophylla&filter=&fuzzy=false&nameType=all&dbs=wcsCmp) ) | Boil decoction in water, twice daily for 15 consecutive days. |
| Jinyinhua Oral Liquid  (China)  Zhang Youli 2020 | Lonicerae Japonicae Caulis (Caprifoliaceae, Lonicera japonica Thunb); | Three times daily and 60 mL each time for 10 consecutive days |
| Bufei Huoxue capsules (BFHX)  (China)  Yuqin Chen 2020 | 40% of Astragali radix (Leguminosae, [Astragalus mongholicus Bunge](https://mpns.science.kew.org/mpns-portal/plantDetail?plantId=661222&query=Astragali+radix&filter=&fuzzy=false&nameType=all&dbs=wcsCmp)); 40% of paeonia lactiflora pall (Ranunculaceae, Paeoniae radix rubra ) and 20% of Psoraleae fructus (Fulse, [Cullen corylifolium (L.) Medik.](https://mpns.science.kew.org/mpns-portal/plantDetail?plantId=747305&query=Psoraleae+fructus&filter=&fuzzy=false&nameType=all&dbs=wcsCmp)) The drug quality standards conform to the regulations of the Chinese Pharmacopoeia (Chinese Pharmacopoeia Commission, 2015). The chemical construction and quality of BFHX capsules were assessed by high performance liquid chromatography (HPLC). | BFHX (Chinese medicine Z20030063, Guangdong Lei Yun Shang Pharmaceutical Co., Ltd. (Yunfu, Guangdong Province, China); batch number 022001; specifications: 0.35 g per capsule) |
| Persian medicine (Iran)  Mehrdad Karimi 2021 | **Capsule 1:** Rheum Palmatum (Polygonaceae, Rheum Palmatum L.); Glycyrrhiza uralensis (Leguminosae, [Glycyrrhiza uralensis Fisch. ex DC.](https://mpns.science.kew.org/mpns-portal/plantDetail?plantId=827776&query=Glycyrrhiza+uralensis&filter=&fuzzy=false&nameType=all&dbs=wcsCmp)); Punica granatum(Punicaceae, Punica granatum L.)  **Capsule 2:** Nigella sativa (Ranunculaceae, Nigella sativa L.)  Decoction:10g of Matricaria chamomilla (Asteraceae ,Matricaria chamomilla L.); 10g of Zataria multiflora (Lamiaceae, Zataria multiflora Boiss.); 10g of Glycyrrhiza uralensis (Leguminosae, [Glycyrrhiza uralensis Fisch. ex DC.](https://mpns.science.kew.org/mpns-portal/plantDetail?plantId=827776&query=Glycyrrhiza+uralensis&filter=&fuzzy=false&nameType=all&dbs=wcsCmp)); 10g of Ziziphus jujuba (Rhamnaceae, Ziziphus jujuba Mill.); 10g of Ficus carica (Moraceae ,Ficus carica L.); 10g of Urtica dioica (Urticaceae, Urtica dioica L.); 10g of Althaea officinalis  (Malvaceae, Althaea officinalis L.) and10g of Nepeta bracteata Benth (Lamiaceae, Nepeta bracteata Benth.) | Capsule 1: first, rhizome of Rheum palmatum L.,  root of G. glabra L., and fruit peel of Punica granatum L. were mixed in a ratio of 0.5:1: 1 and pulverized with an electric mill. They were then extracted by maceration using 4 L of 70% of hydroethanolic solution (70:30 vol/vol ethanol: water) every 48 hr for three times. Then, the extract was separated and concentrated using rotary evaporator as  much as possible. It was put in a vacuum oven until maximum drying.Afterward, lyophilized powder was prepared via a freeze dryer. The final product was mixed with starch (Merck, Germany) as filler and was packaged in 500-mg capsules. It was determined that every capsule has lyophilized powder of hydroalcoholic extract of one-gram raw material of G. glabra and P. granatum and 0.5-g raw material of R. palmatum, and the rest of each capsule was filled by starch as filler andwas packaged in 500mg capsules. . It was determined that every capsule  has lyophilized powder of hydroalcoholic extract of one-gram raw material of G. glabra and P. granatum and 0.5-g raw material of R. palmatum, and the rest of each capsule was filled by starch as filler.  Capsule 2: Seed of Nigella sativa L. was powdered and packaged in 500-mg capsules.  Decoction: Each sachet for one day. one sachet was decocted for 1 hr in 900 cc of water and then filtered and used three times a day (each time about 300 cc). |
| Propolis plus Hyoscyamus niger L. methanolic extract  (Iran)  Razieh Borujerdi 2022 | Hyoscyamus niger (Solanaceae, Hyoscyamus niger L.) | The 10 mL of syrup (containing 1.6 mg of methanolic of Hyoscyamus KOSARI ET AL. 4001niger L extract plus 450 mg of propolis) was administered three times a day to each patient for 6 days. The active ingredients were dissolved in simple syrup BP (British Pharmacopeia) and this simple syrup was also  used as a placebo. |
| Zufa syrup (Iran)  Razieh Borujerdi 2020 | Nepeta bracteata (Lamiaceae, Nepeta bracteata Benth.); Ziziphus jujube (Rhamnaceae, Ziziphus jujuba Mill. ); Glycyrrhiza uralensis (Leguminosae, [Glycyrrhiza uralensis Fisch. ex DC.](https://mpns.science.kew.org/mpns-portal/plantDetail?plantId=827776&query=Glycyrrhiza+uralensis&filter=&fuzzy=false&nameType=all&dbs=wcsCmp); Ficus carica(Moraceae , [Ficus carica subsp. carica](https://mpns.science.kew.org/mpns-portal/plantDetail?plantId=1264461&query=Ficus+carica&filter=&fuzzy=false&nameType=all&dbs=wcsCmp)), Cordia myxa (Boraginaceae, Cordia dichotoma G.Forst.); Papaver somniferum (Papaveraceae, Papaver somniferum L. ); Foeniculum vulgare (Apiaceae, [Foeniculum vulgare Mill.](https://mpns.science.kew.org/mpns-portal/plantDetail?plantId=813604&query=Foeniculum+vulgare&filter=&fuzzy=false&nameType=all&dbs=wcsCmp)); Maidenhair fern(Pteridaceae, Adiantum capillus-veneris L.); Viola tricolor (Violaceae, [Viola arvensis Murray](https://mpns.science.kew.org/mpns-portal/plantDetail?plantId=464083&query=Viola+tricolor&filter=&fuzzy=false&nameType=all&dbs=wcsCmp)); Echium vulgare (Boraginaceae, Echium vulgare L.); Lavandula (Lamiaceae, Lavandula stoechas L. ); Iris tectorum Maxim(Iridaceae, Iris tectorum Maxim) and sugar  The amount of each drug in a polyherbal preparation is unavailable in original text | Zufa syrup (IRC:2129211562044973) was manufactured by Booalidaroo Pharmaceutical Company (Qom, Iran)  Patients were instructed to take 7.5 mL of their syrup every 4 hours for 10 days.  A patent medicine |
| Oral Curcumin with Piperine (India)  Kirti S Pawar 2021 | Curcuma longa (Zingiberaceae,Curcuma longa L.) and Psilocaulon absimile (Aizoaceae, Mesembryanthemum coriarium Burch. ex N.E.Br.) | Treatment group received USFDA-approved Curcumin C3 Complex® (SamiDirect, India) dietary supplement tablets containing 525 mg Tab. Curcumin (diferuloylmethane; 525mg)  with 2.5mg Bioperine® (2.5 mg; SamiDirect) twice a day for14 days. Piperine was used as an adjuvant therapy |
| ImmuActiveTM (India)  Muhammed Majeed 2021 | 100mg of Curcuma longa (Zingiberaceae,Curcuma longa L.); 50mg of Andrographolides(Acanthaceae, Andrographis paniculata); 50mg of Resveratrol; 10mg of Zinc; 40mg of Selenium and 3mg of Piperine(Aizoaceae, Psilocaulon absimile) | ImmuActiveTM was administered orally to subjects once daily after  breakfast in the morning |
| Nilavembu Kudineer and Kaba Sura Kudineer (India)  Anurag Srivastava 2021 | **Nilavembu Kudineer:** Andrographolides (Acanthaceae ,Andrographis paniculata (Burm.f.)); Vetiveria (Poaceae, Vetiveria zizanioides L.); Santalum album (Santalaceae,Santalum album L.); Ginger(Zingiberaceae, Zingiber officinale Roscoe.); Black pepper(Piperaceae,Piper nigrum L.); Cyperus rotundus(Cyperaceae, Cyperus rotundus L.); Hedyotis corymbosa(Rubiaceae, Hedyotis corymbosa L.Lam); Plectranthus (Lamiaceae, Plectranthus vettiveroides); Trichochanthes(Cucurbitaceae, Trichochanthes cucumerina L.)  **Kaba Sura Kudineer** : Ginger(Zingiberaceae, Zingiber officinale Roscoe); Black pepper(Piperaceae, Piper longum L.); Clove(Myrtaceae, Syzygium aromaticum); Pyrethrum(Asteraceae, Anacyclus pyrethrum L.); Tragia involucrate (Euphorbiaceae, Tragia involucrate L.); Solanumanguivi(Solanaceae, Solanumanguivi Lam); Chebulic myrobalan(Combretaceae, Terminalia chebula (Gaertn.)); Malabar nut(Acanthaceae, Justicia adathoda Linn.); Coleus strobilifer (Lamiaceae, Anisochilus carnosus (L.f) Wall), ex Benth(Rubiaceae,Lasianthus chinensis); Cheilocostus speciosus(Costaceae, Costus speciosus (J.Koing)Sm); gurjo(Menispermaceae, Tinospora cordifolia (Thunb.) Miers); Clerodendrum (Lamiaceae,Clerodendrum serratum (L.); Andrographolides (Acanthaceae ,Andrographis paniculata (Burm.f.)); Cyperus rotundus (Cyperaceae,Cyperus rotundus L.); Kurumthotti (Malvaceae, Sida acuta (Burm.f.)  The amount of each drug in a polyherbal preparation is unavailable in original text | In order to obtain NVK or KSK decoctions, a 5-mg coarse powder of NVK or KSK, obtained from the Central Pharmacy-Central Council for Research in Siddha (CCRS), Chennai, India, was boiled in 240 ml of water and reduced to one fourth (60ml), followed by filtration. Participants were randomized to receive 60 ml of placebo in Arm I, 60 ml of NVK in Arm II, and 60 ml of KSK in Arm III, twice a day post morning and evening meals  https://doi.org/10.1016/j.jaim.2020.05.009. https://pubmed.  ncbi.nlm.nih.gov/32527713/. |
| BNO 1030(Ukraine)  Vasyl Popovych 2021 | 0.4g of Marshmallow root (Malvaceae , Radix Althaeae); 0.3g of Cammomile flowers (Asteraceae, Flores Chamomillae); 0.5g of Horstail herb(Equisetaceae, Herba Equiseti); 0.4g of Walnut leaves(Juglandaceae, Folia Jungladis); 0.4g of Yarrow herb (Asteraceae, Herba Millefolii); 0.2g of Oak bark (Fagaceae, Cortex Quercus); 0.4g of Dandelion herb(Asteraceae ,Herba Taraxaci) | BNO 1030 (Imupret®) drops for oral administration, from one batch, in the following dosages: 25 drops 6 times a day.  BNO 1030 drops for oral administration are a standardized aqueous-alcoholic extract. Active substances:100 g drops contain 29 g of an alcoholic aqueous extract (extracting agent: ethanol 59 % (V/V) made from the following  medicinal plants: |
