# Supplementary material for: Therapeutic effects of herbal-medicine combined therapy for COVID-19: A systematic review and meta-analysis of randomized controlled trials
Source: Front Pharmacol. 2022 Sep 1;13:950012. doi: 10.3389/fphar.2022.950012 (PMC9475194; doi:10.3389/fphar.2022.950012)
Supplement: Supplementary file 4 [file DataSheet1.pdf]

Appendix 3 Random effect model of chest CT and fatigue improvement

Study or Subgroup	Combination Events	Combination Total	Control Events	Control Total	Weight	Risk Ratio M-H, Random, 95% CI
2020 Ding	32	51	21	49	3.5%	1.46 [0.99, 2.15]
2020 Jin	17	18	16	20	6.6%	1.18 [0.92, 1.51]
2020 Lu	12	22	5	23	0.8%	2.51 [1.06, 5.95]
2020 Qiu	5	25	2	25	0.3%	2.50 [0.53, 11.70]
2020 Sun	32	32	18	25	6.5%	1.38 [1.08, 1.77]
2020 Wang JB	21	24	16	23	4.8%	1.26 [0.92, 1.71]
2020 Wang L	37	40	32	40	9.2%	1.16 [0.97, 1.38]
2020 Wang LQ	52	58	52	60	11.5%	1.03 [0.91, 1.18]
2020 Zeng	93	104	89	125	11.6%	1.26 [1.10, 1.43]
2020 Zhang YL	80	80	37	40	13.5%	1.09 [0.99, 1.19]
2020 Zhu	9	25	8	25	1.0%	1.13 [0.52, 2.44]
2021 Chao	46	51	34	45	8.7%	1.19 [0.99, 1.44]
2021 Ho	29	34	13	30	2.9%	1.97 [1.28, 3.03]
2021 Hu K	119	142	91	142	10.9%	1.31 [1.13, 1.51]
2021 Liu	69	99	55	96	7.6%	1.22 [0.98, 1.51]
2022 Chen	8	53	4	51	0.5%	1.92 [0.62, 6.00]
**Total (95% CI)**		**858**		**819**	**100.0%**	**1.23 [1.13, 1.33]**
Total events	661		493			

Heterogeneity: Tau² = 0.01; Chi² = 29.18, df = 15 (P = 0.02); I² = 49%
Test for overall effect: Z = 4.96 (P < 0.00001)

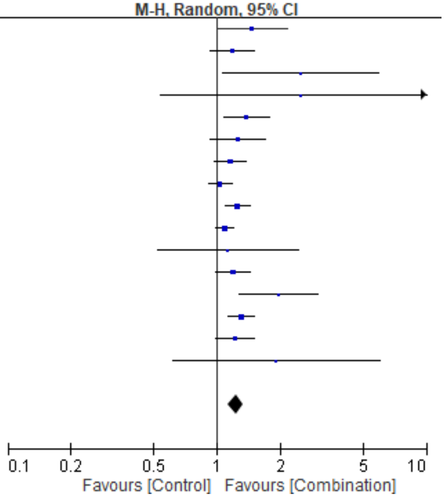

Figure 5a: comparison of HM combined therapy for chest CT manifestations in COVID19 patients using random effect model

Study or Subgroup	Combination Events	Combination Total	Control Events	Control Total	Weight	Risk Ratio M-H, Random, 95% CI
2020 AI	9	10	5	12	2.7%	2.16 [1.07, 4.35]
2020 Duan	45	58	14	26	7.4%	1.44 [0.98, 2.11]
2020 Jin	12	18	10	20	4.2%	1.33 [0.77, 2.30]
2020 Lin	26	34	3	7	1.8%	1.78 [0.74, 4.28]
2020 Sun	14	14	8	10	8.9%	1.25 [0.90, 1.75]
2020 Wang LQ	32	58	21	60	6.5%	1.58 [1.04, 2.39]
2020 Xiao	45	53	41	50	17.6%	1.04 [0.87, 1.23]
2020 Xiong	15	19	6	14	3.1%	1.84 [0.96, 3.52]
2020 Zhang YL	53	58	18	21	16.2%	1.07 [0.88, 1.29]
2021 An	56	65	16	19	14.5%	1.02 [0.82, 1.27]
2021 Liu	72	99	66	96	17.0%	1.06 [0.88, 1.27]
**Total (95% CI)**		**486**		**335**	**100.0%**	**1.18 [1.05, 1.34]**
Total events	379		208			

Heterogeneity: Tau² = 0.01; Chi² = 16.50, df = 10 (P = 0.09); I² = 39%
Test for overall effect: Z = 2.71 (P = 0.007)

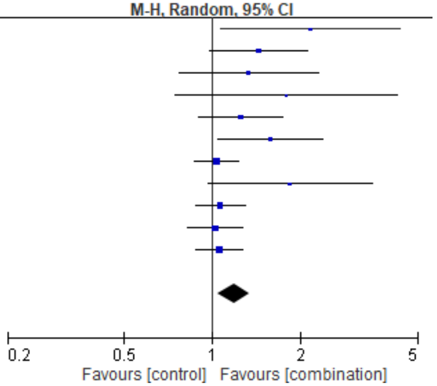

Figure 5b: comparison of HM combined therapy for fatigue reduction cases in COVID19 patients using random effect model
